# SMAD3 Deficiency Promotes Inflammatory Aortic Aneurysms in Angiotensin II–Infused Mice Via Activation of iNOS

**DOI:** 10.1161/JAHA.113.000269

**Published:** 2013-06-21

**Authors:** Chek K. Tan, Eddie H. Tan, Baiwen Luo, Charlotte L. Huang, Joachim S. Loo, Cleo Choong, Nguan S. Tan

**Affiliations:** 1 School of Biological Sciences, Nanyang Technological University, Nanyang, Singapore (C.K.T., E.H.T., N.S.T.); 2 School of Materials Science and Engineering, Nanyang Technological University, Nanyang, Singapore (B.L., C.L.H., J.S.L., C.C.); 3 Institute of Molecular and Cell Biology, A*STAR, Proteos, Singapore (N.S.T.)

**Keywords:** aneurysm, inflammation, iNOS, SMAD3, TGF‐β

## Abstract

**Background:**

Ninety percent of the patients carrying distinct *SMAD3* mutations develop aortic aneurysms and dissections, called aneurysms‐osteoarthritis syndrome (AOS). However, the etiology and molecular events downstream of SMAD3 leading to the pathogenesis of aortic aneurysms in these patients still remain elusive. Therefore, we aimed to investigate the vascular phenotypes of *SMAD*3‐knockout mice.

**Methods and Results:**

We have shown that angiotensin II–induced vascular inflammation, but not hypertension, leads to aortic aneurysms and dissections, ultimately causing aortic rupture and death in mice. Lipopolysaccharide‐triggered inflammation confirmed that enhanced aortic macrophage recruitment was essential for aneurysm formation in angiotensin II–infused *SMAD3*‐knockout mice. In contrast, phenylephrine‐triggered hypertension alone was insufficient to induce aortic aneurysms in mice. Using uniaxial tensile and contractility tests, we showed that SMAD3 deficiency resulted in defective aortic biomechanics and physiological functions, which caused weakening of the aortic wall and predisposed the mice to aortic aneurysms. Chromatin immunoprecipitation (ChIP) and re‐ChIP assays revealed that the underlying mechanism involved aberrant upregulation of inducible nitric oxide synthase (iNOS)–derived nitric oxide production and activation of elastolytic matrix metalloproteinases 2 and 9. Administration of clodronate‐liposomes and iNOS inhibitor completely abrogated these aortic conditions, thereby identifying iNOS‐mediated nitric oxide secretion from macrophages as the downstream event of SMAD3 that drives this severe pathology.

**Conclusions:**

Macrophage depletion and iNOS antagonism represent 2 promising approaches for preventing aortic aneurysms related to *SMAD3* mutations and merit further investigation as adjunctive strategies for the life‐threatening manifestations of AOS.

## Introduction

Aortic aneurysms and dissections are 2 major diseases affecting the aorta, accounting for ≈53 000 deaths worldwide annually.^1^ Aortic aneurysm is defined as an abnormal widening of a portion of the aorta due to weakness in the vascular wall as a result of the normal aging process or genetic mutations. Aortic aneurysms are typically indolent and asymptomatic unless a rupture occurs, thus rendering a poor prognosis to the individuals suffering from this disease. The rupture of an aneurysmal aorta represents the end stage of the disease and leads to life‐threatening hemorrhage with a poor survival rate. Despite its cost and high rates of associated morbidity and mortality, surgery remains the only intervention for individuals with aortic aneurysms. Currently, there is no approved drug treatment for this disease. Therefore, a better understanding of the pathophysiology will accelerate the development of novel therapeutic strategies and management procedures.

SMAD3 is a transcription factor involved in the canonical signaling of transforming growth factor (TGF)‐β, which plays a central role in connective tissue turnover and homeostasis, tissue remodeling, and fibrosis.^2^ The phosphorylation of SMAD3 by cell‐surface TGF‐β receptors results in the recruitment of SMAD4, its translocation into the nucleus, and the transcription of SMAD‐dependent genes. *SMAD3* mutations have recently been identified as a cause of familial thoracic aortic aneurysms and dissection with intracranial and other arterial aneurysms.^3^ Approximately 90% of the patients carrying any of the 8 distinct *SMAD3* mutations develop aortic aneurysms and dissections.^4–5^ These aortic anomalies represent the vascular manifestations of aneurysms‐osteoarthritis syndrome (AOS), which is a multisystem connective tissue disorder characterized by the presence of arterial aneurysms and tortuosity, along with early‐onset osteoarthritis.^4^ However, the events downstream of SMAD3 leading to the pathogenesis of aortic aneurysms remain unknown. *SMAD3*‐knockout (S3KO) mice exhibit early‐onset osteoarthritis closely resembling the joint abnormalities observed in human AOS.^4,6^ Thus, using these mice as an experimental model for elucidating the underlying mechanisms of aortic aneurysm in AOS is justifiable. In this study, we aimed to study the aortic phenotype of these mice.

## Methods

### Antibodies

Antibodies against CCAAT/enhancer binding protein (C/EBP)–β (#sc‐150), p300 (#sc‐585), α‐smooth muscle actin (α‐SMA [#sc‐69972]), β‐tubulin (#sc‐9104), horseradish peroxidase (HRP)–conjugated goat anti‐mouse IgG (#sc‐2005), and preimmune serum (#sc‐2338) were purchased from Santa Cruz Biotechnology. Antibody against matrix metalloproteinase (MMP)–9 (#ab38898) was obtained from Abcam. Antibodies against types I (#234167) and III (#234189) collagen, inducible nitric oxide synthase (iNOS/NOS II [ABN26]), elastin (#MAB2503), and HRP‐conjugated goat anti‐rabbit IgG (#AP187P) were purchased from Millipore. Rat anti‐mouse F4/80 antibody (#MCA497) was obtained from AbD Serotec. Antibodies against total and phosphorylated nuclear factor‐kappaB (NF‐κB), #4764 and #3033, respectively, were purchased from Cell Signaling Technology. Rat anti‐mouse CD68 antibody (#137001) was acquired from Biolegend. All other fluorophore‐conjugated secondary antibodies were acquired from Life Technologies.

### Animals

Colonies of wild‐type (WT) and S3KO mice on a C57BL/6J×Sv129 mixed background were bred in our animal facility by intercrossing mice heterozygous for the targeted disruption. S3KO mice were initially generated by targeted disruption of exon 8 of the *SMAD3* gene using a homologous recombination technique as previously described.^7^ The truncated portion of the gene is at the SMAD3 C‐terminal end that contains a SSVS consensus phosphorylation site and a L3 loop, which are essential for the interaction with TGF‐β receptors. S3KO mice have been shown to be functionally null for SMAD3‐mediated signaling.^7^ Mice were maintained at 25°C on a 12‐/12‐hour light‐dark cycle in a specific pathogen‐free facility and routinely monitored. All animals were kept in microisolator cages and given standard rodent chow diet and water ad libitum. Mice were euthanized by CO_2_ asphyxiation. All experimental protocols were approved by the Institutional Animal Care and Use Committee (ARF‐SBS/NIE‐A0167‐AZ) in Singapore. Only age‐matched males were used in all experiments avoid estrous cycle variations that might introduce confounding factors in data interpretation.

### Drug Infusions and Treatments

Angiotension II (AngII; #A9525; Sigma‐Aldrich) was infused in 8‐week‐old mice via subcutaneous osmotic pumps (model 2004; Alzet) at 500 ng/kg per minute (low dose) or 1000 ng/kg per minute (high dose) for 28 days. Lipopolysaccharide (LPS) (#L3012; Sigma‐Aldrich), which was extracted from *Escherichia coli* (serotype O111:B4) and purified by gel filtration, was administered intravenously at 1 mg/kg per week for 4 weeks. This LPS serotype has been used to induce iNOS in human hepatocytes.^8^ Phenylephrine (PE; #P6126; Sigma‐Aldrich) was infused in mice via subcutaneous osmotic pumps at 0.15 mg/kg per day as previously described.^9^ The combined administration of LPS and PE was performed at the concentrations mentioned above. The β‐blocker pindolol (#P0778; Sigma‐Aldrich) was administered in the high‐dose AngII‐infused mice via their drinking water at a concentration of 0.67 mg/mL. We previously showed that water consumption did not differ between WT and S3KO mice.^10^ For the vehicle control, saline was infused or injected in mice instead. For insertion of the osmotic pump, a 1‐cm transverse midscapular incision was made on the dorsal skin of an anesthetized mouse. Subsequently, a sterile hemostat was inserted into the incision to create a pocket by opening and closing the jaws of the hemostat. The pocket was created large enough to allow some free movement of the pump (eg, 1 cm longer than the pump). The vehicle‐ or AngII‐filled pump was then inserted into the pocket with the delivery portal facing away from the incision. The incision was closed with two 7‐mm Reflex wound clips (Alzet) and allowed to heal.

### Blood Pressure Measurements

Mean systolic blood pressure was measured weekly in conscious mice by volume pressure recording using a tail‐cuff method (CODA 8‐Channel High Throughput Non‐Invasive Blood Pressure System, Kent Scientific Corporation) as previously described.^11^ Briefly, mice were secured in a restrainer magnetically attached to a metal platform heated to 35°C. Mice were acclimatized to the setting for 5 minutes. A tail cuff was used to constrict caudal artery flow, and photoelectric sensors detected the tail pulses as cuff pressure was reduced. Systolic blood pressure was calculated as the average of 10 measurements for each mouse. Measurements were performed before drug treatments (day 0) and once weekly for 4 weeks thereafter.

### Histological and Morphometric Analyses of Aorta

After mice were sacrificed by CO_2_ asphyxiation, laparotomy was immediately performed, followed by transection at the inferior vena cava to create an opened circulatory system. Residual blood in the aortas was flushed out by infusion of Krebs–Henseleit buffer (pH 7.4, gassed with 95% O_2_ and 5% CO_2_ for 30 minutes) containing 119 mmol/L NaCl, 1.2 mmol/L MgCl_2_, 1.2 mmol/L NaH_2_PO_4_, 15 mmol/L NaHCO_3_, 4.6 mmol/L KCl, 1.5 mmol/L CaCl_2_, and 11 mmol/L glucose through the left ventricle. The aortas were excised, denuded of periaortic fat, embedded in OCT tissue‐freezing medium (Leica Microsystems), and frozen in liquid nitrogen and stored at −20°C. Fresh‐frozen specimens were sectioned at 8‐μm thickness, fixed in 4% paraformaldehyde dissolved in phosphate‐buffered saline (PBS), pH 7.4, at room temperature for 10 minutes, and stained with hematoxylin and eosin. Images were captured using a Nikon Eclipse 90i bright‐field microscope with a 20× objective lens and QCapture Pro software (QImaging). Morphometric measurements of WT and S3KO aorta were performed using Image J (version 1.41o, NIH). Morphometric parameters were compared between WT and S3KO aortas of the same anatomical locations after normalization to body length (mouth‐to‐anus length) or body surface area.

### Quantification and Characterization of Aneurysms

Quantification of aneurysms was based on percent incidence as previously described.^12^ The severity of aortic aneurysms was based on a previously described classification^13^ in which aortic aneurysms were assigned to a category dependent on the gross phenotype of the tissue. Aneurysms were scored based on the following scale: stage I, the presence of remodeled tissue that frequently contains thrombus; stage II, a pronounced bulbous form of stage I that contains thrombus; stage III, a form in which multiple aneurysms containing thrombus were present; stage IV, attributed to ruptured aneurysms. Aneurysm incidence and severity were independently determined by 2 unblinded observers. There was complete concordance of designation by the 2 observers.

### Histological Analysis of Spleen

Fresh spleen samples were excised and weighed. The weights were then normalized to the body weight of mice. The whole spleens were subsequently processed, embedded, and sectioned using methods similar to that for the aorta. Systemic inflammation was determined by the presence of germinal centers within the white pulps. Germinal centers are sites in which mature B‐lymphocytes undergo proliferation, differentiation, and somatic hypermutation in response to infection. The number and total area of germinal centers in spleen sections were calculated using ImageJ software and normalized to the sectional area in square millimeter and total spleen sectional area, respectively.

### Isolation of Primary White Blood Cells, Peritoneal Macrophages, and Aortic Vascular Smooth Muscle Cells

Whole blood was isolated from mice via cardiac puncture and collected in BD Vacutainer containing K2 ethylenediaminetetraacetic acid (EDTA) to prevent coagulation. Thereafter, 10 mL of sterile red blood cell lysis buffer containing 155 mmol/L NH_4_Cl, 10 mmol/L KHCO_3_, and 0.1 mmol/L EDTA was added to the blood samples and incubated for 5 minutes at room temperature. After red blood cell lysis, an equal volume (≈12 mL) of ice‐cold PBS was added to the suspension, followed by centrifugation at 400*g* at 4°C for 5 minutes to pellet the WBCs for flow cytometric analysis. For isolation of resident peritoneal cells, mice were euthanized by CO_2_ asphyxiation to avoid excessive bleeding into the peritoneal cavity. Because minimal aortic macrophages were detectable in untreated mice according to the F4/80 staining of aortic sections, the peritoneal cavity provided an alternative and easily accessible site for harvesting moderate numbers of resident macrophages. Generally, macrophages isolated from the mouse peritoneal cavity are mature quiescent macrophages.^14–15^ Peritoneal macrophages were isolated via peritoneal flush using a method previously described.^16^ Briefly, 5 mL of ice‐cold PBS was injected into the peritoneal cavity by inserting a 20‐gauge needle through the peritoneal wall along the mouse's left side (spleen side). Using the same syringe and needle, the fluid was aspirated from the peritoneum and collected into a 50‐mL conical polypropylene centrifuge tube on ice. The peritoneal exudate cells were then centrifuged at 400*g* at 4°C for 5 minutes to pellet the cells for flow cytometric analysis. Vascular smooth muscle cells (VSMCs) were isolated from the murine aorta based on the method previously described.^17^ Briefly, the whole aorta was dissected out from its origin at the left ventricle to the iliac bifurcation, denuded of periaortic fat, cut into small pieces (≈2‐mm segments), and incubated in 5 mL of Dulbecco's modified Eagle's medium (DMEM) containing 10% fetal bovine serum (#16000036; Life Technologies) and 1.36 mg/mL of type II collagenase (#LS004174; Worthington Biochemical Corporation) in agitation at 115 rpm for 2 hours at 37°C. After digestion, 5 mL of medium was added, and the cells were centrifuged at 300*g* at 4°C for 5 minutes to pellet the cells for flow cytometric analysis.

### Flow Cytometry

For determination of systemic inflammation, total WBCs isolated from the whole blood were resuspended in 400 μL of staining buffer (PBS+3% fetal bovine serum) containing CD68 antibody (1:400) and incubated for 30 minutes on ice. Subsequently, cells were washed twice with the staining buffer and centrifuged at 400*g* at 4°C for 5 minutes. Cells were then resuspended in 400 μL of staining buffer containing the secondary antibody (1:400) and incubated on ice for another 30 minutes, followed by 2 washes and flow cytometric analysis. For determination of the expression of MMP‐9 and iNOS in peritoneal macrophages, isolated peritoneal exudate cells were incubated with CD68 and secondary antibodies as mentioned above before fixation and permeabilization in 100 μL of BD Cytofix/Cytoperm Buffer (BD Biosciences) for 30 minutes at room temperature, followed by washing with 1 mL of BD Perm/Wash Buffer and incubation with BD Cytoperm Permeabilization Buffer Plus for 10 minutes on ice. Cells were then refixed with 100 μL of BD Cytofix/Cytoperm Buffer for another 5 minutes on ice. Intracellular staining for MMP‐9 or iNOS was carried out by incubating the fixed and permeabilized cells with staining buffer containing the MMP‐9 or iNOS antibody (1:400) for 30 minutes on ice, followed by incubation with the respective secondary antibody for another 30 minutes on ice before flow cytometric analysis. For determination of the expression of MMP‐9 and iNOS in aortic VSMCs, isolated cells were directly fixed and permeabilized as mentioned above, followed by concurrent incubation of α‐SMA antibody (1:400) and MMP‐9 or iNOS antibody. Flow cytometric analysis was performed using the BD Accuri C6 Flow Cytometer (BD Biosciences) equipped with lasers with excitation wavelengths of 488 and 640 nm and optical filters for detecting fluorescence wavelengths at 533±30 nm (FL1) and 675±25 nm (FL4). The fluorescence gates were generated on the basis of the single positive controls. Cellular expression of MMP‐9 and iNOS was determined on the basis of the 10 000 events of either CD68^+^ (a monocyte/macrophage marker) or α‐SMA^+^ (a VSMC marker) cells acquired. Analysis was subsequently performed using FlowJo software (version 10.0.5; Tree Star Inc).

### Total RNA Extraction and Reverse Transcription

Aortic tissue denuded of periaortic fat was homogenized in 1 mL of TRIzol Reagent (Invitrogen), followed by incubation of the homogenate at room temperature for 5 minutes to allow complete dissociation of nucleoprotein complexes. For each milliliter of TRIzol Reagent, 200 μL of chloroform was added and mixed by shaking the tube vigorously for 15 seconds. Subsequently, the tube was incubated at room temperature for 3 minutes. The samples were then centrifuged at 12 000*g* for 15 minutes at 4°C to segregate the mixture into a lower red phenol–chloroform phase, an interphase, and a colorless upper aqueous phase containing the RNA molecules. Approximately 400 μL of the colorless aqueous phase was transferred to a fresh tube, where an equal volume of 70% ethanol was added and mixed by vortexing. The extracted RNA was further purified using E.Z.N.A. Total RNA Kit I (Omega Bio‐tek) according to the manufacturer's protocol. The purified RNA was spectrophotometrically quantified, and its quality was assessed by measuring the absorbance ratios at 260/280 and 260/230 nm using a Nanodrop Spectrophotometer (Thermo Fisher Scientific). One microgram of total RNA was reverse‐transcribed with iScript Reverse Transcription Supermix (Bio‐Rad), which contains MMLV (RNaseH^+^)–reverse transcriptase, RNase inhibitor, dNTPs, oligo(dT), random primers, buffer, and MgCl_2_. Priming was carried out at 25°C for 5 minutes, followed by reverse transcription at 42°C for 30 minutes and reverse transcriptase inactivation at 85°C for 5 minutes.

### Real‐Time Quantitative Polymerase Chain Reaction

Real‐time quantitative polymerase chain reaction (RT‐qPCR) was performed as previously described.^10^ Briefly, 3 sets of tissue samples were used for each genotype. The sequence of the primer pairs was obtained from the PrimerBank database, available online at http://pga.mgh.harvard.edu/primerbank/ and summarized in Table 1. RT‐qPCR was performed on a CFX96 Real‐Time PCR Detection System (Bio‐Rad) equipped with CFX Manager Software using a Kapa Sybr Fast qPCR kit (Kapa Biosystems).

**Table 1.** Sequences of Primers Used in this Study

Gene Name	GenBank Accession ID	Primer Sequence (5′→3′)
Gene expression study		
AT1a	NM_177322	Reverse (CGTGCTCATTTTCGTAGACAGG)
		Forward (TGCCATGCCCATAACCATCTG)
AT1b	NM_175086	Reverse (GAAGGGCGGTAGGAAAGAGTA)
		Forward (CTGTGAAATTGCGGACGTAGT)
AT2	NM_007429	Reverse (AAGGGTAGATGACCGATTGGT)
		Forward (ATGATTGGCTTTTTGGACCTGT)
IL‐6	NM_031168	Reverse (TTGGTCCTTAGCCACTCCTTC)
		Forward (TAGTCCTTCCTACCCCAATTTCC)
iNOS	NM_010927	Reverse (TAGCCAGCGTACCGGATGA)
		Forward (CAGCACAGGAAATGTTTCAGC)
MCP‐1	NM_011333	Reverse (GCATTAGCTTCAGATTTACGGGT)
		Forward (TTAAAAACCTGGATCGGAACCAA)
MMP‐2	NM_008610	Reverse (CCATCAAACGGGTATCCATCTC)
		Forward (TTTGCTCGGGCCTTAAAAGTAT)
MMP‐9	NM_013599	Reverse (AGCTCGGTGGTGTTCTCCAATG)
		Forward (AAACCACCTCTCCCGACTCCAG)
TIMP‐1	NM_011593	Reverse (ACCTGATCCGTCCACAAACAG)
		Forward (CTTGGTTCCCTGGCGTACTC)
TIMP‐2	NM_011594	Reverse (GGGAGGAGATGTAGCAAGGG)
		Forward (ACACGCTTAGCATCACCCAG)
ChIP and Re‐ChIP		
iNOS promoter_C/EBP binding site (−515 to −318 bp)	NM_010927.3	Reverse (TTAGCTCATTCATGATGGACACTCC)
		Forward (TGCAAGCCAGGGTATGTGGTTTAG)
iNOS promoter_Control (−1796 to −1604)	NM_010927.3	Reverse (ACAGGAATATGCCTAGGGTTTGC)
		Forward (TATGCTGAAATCCATAAGCTGTGTG)

AT indicates angiotensin; IL, interleukin; iNOS, inducible nitric oxide synthase; MCP, monocyte chemotactic protein; MMP, matrix metalloproteinase; TIMP, tissue inhibitor of metalloproteinase; EBP, enhancer binding protein.

### Immunofluorescence Staining

Fresh aorta tissue sections were fixed in 100% acetone at −20°C for 5 minutes and washed twice with 1× PBS. Tissue sections were either blocked with 2% bovine serum albumin (BSA) containing 0.1% Triton X‐100 (ie, elastin and collagen staining) or 5% normal goat serum (NGS); that is, α‐SMA, iNOS, and F4/80 staining, for 1 hour, followed by incubation with the respective primary antibodies (1:100) overnight at 4°C in either 2% BSA or 5% NGS. After 3 washes with PBS, sections were incubated for 1 hour at room temperature with the respective secondary antibodies (1:250), followed by 3 washes with 1× PBS and mounting with DAPI‐mounting medium. For double immunofluorescence staining for MMP‐9 and α‐SMA or F4/80, tissue sections were fixed as described above, followed by an antigen retrieval procedure in 10 mmol/L sodium citrate buffer (pH 6.0) at 95°C for 20 minutes. Next, sections were incubated with blocking/permeabilization buffer (PBS+2% BSA+1% Triton‐X+0.05% Tween‐20), followed by concurrent incubation of primary antibodies (F4/80 [1:500]+MMP‐9 [1:1000] or α‐SMA [1:100]+MMP‐9 [1:1000]) overnight at 4°C. The next day, sections were washed as described above, incubated with the respective secondary antibodies, and mounted with VectaShield DAPI mounting medium (Vector Laboratories). Images were captured using a confocal microscope (Carl Zeiss) with a 40× or 63×/1.40 oil objective equipped with a AxioCam MRm camera (Carl Zeiss) and analyzed using ZEN 2009 software (Carl Zeiss).

### Scanning Electron Microscopy

Tissue preparation for scanning electron microscopy of internal elastic lamella and collagen fibers was performed as previously described with minor modifications.^18–19^ For scanning electron microscopy of internal elastic lamella, aortic rings extracted from the thoracic and abdominal aorta were dissected immediately after animal euthanization with CO_2_ asphyxiation. The aortic segments were then cut open and immersed in 0.1 N NaOH at 80°C for 1 hour to decellularize the aorta, after which they were successively transferred into 0.1 N NaOH at room temperature for 5 minutes, into distilled water at room temperature for 5 minutes, into 0.1 N HCl at room temperature for 2 minutes to remove collagen, and finally into a saline solution for 2 hours. The aortic segments were then fixed in 2% glutaraldehyde and 1% paraformaldehyde in 0.1 mol/L PBS (pH 7.4) overnight at 4°C. Following fixation, the tissues were washed twice with PBS and postfixed in 1% osmium tetroxide for 1 hour. The specimens were then dehydrated in graded concentrations of ethanol ranging from 50% to 100% and dried in a critical‐point drying apparatus using liquid CO_2_ as immersion medium. The samples were then mounted onto a metal stub with double‐sided carbon tape. After this, the specimens were sputter‐coated with a thin layer of gold using SPI‐Module Sputter Coater System (Structure Probe) and examined with a JSM‐5410LV scanning electron microscope (JEOL). For scanning electron microscopy of collagen fibers, tissues were decellularized as described above, and elastic fibers were removed from the decellularized aorta by elastase treatment at 37°C for 30 minutes at a concentration of 9.5 U/mL in 100 mmol/L Tris buffer (pH 7.4) containing 1 mmol/L CaCl_2_ and 0.02% NaN_3_.

### Protein Extraction and Western Blot Analysis

Protein was extracted from the whole aorta denuded of periaortic fat by grinding the tissue using a rotor‐stator homogenizer in ice‐cold lysis buffer that contained 20 mmol/L Na_2_H_2_PO_4_, 250 mmol/L NaCl, 1% Triton X‐100, 0.1% sodium dodecyl sulfate (SDS), 1 mmol/L Na_3_VO_4_, and 1× proteinase inhibitor cocktail. After homogenization, cell lysate was centrifuged at 13 000*g* at 4°C for 10 minutes to sediment the cell debris. For Western blot analysis, an equal amount of protein extracts was resolved on a 10% SDS‐polyacrylamide gel and electrotransferred onto a polyvinylidene fluoride membrane. Membranes were blocked with 5% milk dissolved in Tris Buffer Saline with 0.1% Tween 20 (TBST) for 1 hour at room temperature, followed by primary antibody incubation overnight at 4°C at a dilution range of 1:2000 to 1:3000 in 5% milk or BSA dissolved with TBST. After washing 3 times with TBST for 5 minutes each, HRP‐conjugated secondary antibodies were added at a dilution of 1:5000 in 5% milk/TBST, and the membranes were incubated for 1 hour at room temperature. Chemiluminescence was detected by incubating the membranes with Luminata Crescendo Western HRP Substrate (Millipore) at room temperature for 5 minutes. Beta‐tubulin was used to check for equal loading and transfer. Band intensity for each blot was subsequently determined by Image J.

### Terminal Deoxynucleotidyl Transferase dUTP Nick End Labeling Assay

Fresh aortic tissues were processed, embedded, and sectioned as described above. Sections were then fixed in 4% paraformaldehyde/PBS (pH 7.4) at room temperature for 20 minutes, followed by washing with PBS twice for 15 minutes each. Antigen retrieval was performed by incubating the sections with 0.1% Triton X‐100/10 mmol/L Tris at room temperature for 8 minutes in a dark humid chamber. The sections were then rinsed with PBS twice for 5 minutes each. Staining for DNA fragmentation that results from apoptosis was performed using an In Situ Cell Death Detection Kit, Fluorescein (Roche Diagnostics) as per the manufacturer's protocol. Briefly, sections were incubated with labeling and enzymatic solution (10:1) at 37°C for 1 hour in a dark humid chamber, followed by rinsing with PBS twice for 5 minutes each. The sections were then mounted with DAPI mounting medium. Images were captured as described above. The number of apoptotic cells was counted using ImageJ and expressed as the percentage of the total number of cells, which was determined by counting the cells positively stained for DAPI.

### Uniaxial Tensile Test

The whole aorta was fixed to the tensile testing machine (model 5567; Instron) with rubber‐coated grips equipped with a load cell capacity of 10 N, submerged in PBS that was preheated to 37°C, and pulled at a rate of 1 mm/min until failure. A stress–strain curve reflecting the relationship between force and extension was generated using Bluehill software, version 3.00 (Instron). The biomechanical parameters were measured from the curves generated using Image J.

### Circumferential Tensile and Contractility Tests

The descending thoracic aorta was isolated and cleaned of periaortic fat, connective tissues, and blood, with special care taken to preserve the endothelium. A segment of the aorta (2 mm) was then mounted isometrically in a small vessel myograph (model 410A; Danish Myotechnology) for measuring generated force. The tests were carried out according to previously described protocols.^20^ For circumferential tensile tests, “vessel elasticity” was deduced from the stress–strain curves. In the small vessel myograph, a 2‐mm segment of descending thoracic aorta was stretched by increasing the distance between the 2 stainless wires (=increase in length of VSMC) and held at each length for 3 minutes. Initially, 2 wires were adjusted to L_0_, at which the vessel was not stretched. The inside circumference of the aortic segment was measured as twice the distance between the 2 wires plus the wire circumference plus 2 wire radii (2×20 μm). The distance between the 2 wires was then increased by 100 μm, and the new length was denoted as “L.” The developed force (mN) was divided by the surface area (=inside circumference of the segment×length of the segment) of the aorta segment (mm^2^) to calculate the wall stress (mN/mm^2^). The procedure was repeated until the vessel was unable to maintain its tone. The stress at which rupture occurred was reported as “breaking stress.” The ∆L/L_0_ and the wall stress were fitted on an exponential curve. “Passive force” was measured by repeating the above procedures in a calcium‐free Krebs–Henseleit buffer solution prepared by replacing CaCl_2_ with 320 μmol/L ethylene glycol tetraacetic acid to eliminate VSMC contractility. “Total force” was determined by assessing the active contractility at each level of stretch in response to depolarization (80 mmol/L KCl). For contractility test, aortic segments were stretched to a tension of 1 g. The vessels were thereafter challenged twice with 80 mmol/L KCl, then contracted with PE ranging from 1 nmol/L (10^−9^ mol/L) to 1 mmol/L (10^−3^ mol/L) or AngII ranging from 1 pg/mL to 100 ng/mL. The percent of contraction compared with the maximal contraction induced by incubation with 80 mmol/L KCl was recorded at different concentrations of PE or AngII, and concentration‐response curves were constructed. The negative logarithm (pD_2_) of the concentration of PE giving half‐maximum response (EC_50_) was assessed by linear interpolation on the semilogarithm concentration‐response (pD_2_=−log[EC_50_]). To study the effect of Nω‐nitro‐l‐arginine methyl ester (l‐NAME) and aminoguanidine, either drug was added to the buffer at a concentration of 200 μmol/L and incubated with the aortic segments for 30 minutes before continuing with the protocol.

### Nitric Oxide Staining

Unfixed fresh aortic sections were incubated with 10 μmol/L of 4‐amino‐5‐methylamino‐2′,7′‐difluorofluorescein (DAF‐FM) diacetate (#D‐23844; Molecular Probes) for 1 hour at room temperature, followed by 3 washes with PBS, and were mounted with 10% glycerol/PBS. Images were all taken at the same anatomical location for comparisons between WT and S3KO mice. Relative fluorescence intensity of NO staining of WT and S3KO aorta was measured using Image J.

### In Vivo Chromatin Immunoprecipitation and Re‐ChIP Assays

Whole aortas were excised and stripped clean of adherent fat tissues, followed by mincing the tissues into 3‐ to 4‐mm pieces and immersing in 5 mL of digestion solution (pH 7.3) containing 1000 U activity/mL of type I collagenase (Invitrogen), 50 μg/mL of porcine pancreatic elastase, 0.1 mol/L 4‐(2‐hydroxyethyl)‐1‐piperazineethanesulphonic acid (HEPES), 0.12 mol/L NaCl, 50 mmol/L KCl, 5 mmol/L d‐glucose, 1.5% BSA, and 5 mmol/L CaCl_2_. Subsequently, the minced tissues were digested at 37°C in agitation at 115 rpm for 1 hour. After digestion, cells were quickly washed with PBS and then fixed in 1% formaldehyde at 37°C for 15 minutes. After fixation, glycine was added to a final concentration of 125 mmol/L to stop the crosslinking process. The cells were then pelleted at 1000*g* for 5 minutes and rinsed twice with 10 mL of ice‐cold PBS, followed by resuspending the cells in 1.5 mL of lysis buffer containing 50 mmol/L HEPES‐KOH (pH 7.5), 140 mmol/L NaCl, 1 mmol/L EDTA (pH 8.0), 1% Triton X‐100, 0.1% sodium deoxycholate, 0.1% SDS, and protease and deacetylase inhibitors. To obtain chromatin fragments between 300 and 500 bp, cell lysate was sonicated 7 times for 15 seconds each with 30‐seconds intervals on ice using a Bronson sonifier. After sonification, cell debris was pelleted by centrifugation at 8000*g* for 30 seconds at 4°C. The supernatant was collected for subsequent analysis. For DNA quantification and normalization for qRT‐PCR, 50 μL of the sonicated product was collected (ie, input), and the rest was subjected to ChIP and re‐ChIP as previously described.^21^ Briefly, protein A agarose beads were added to the supernatant and incubated with gentle rocking and rotation for 1 hour. Next, the beads were centrifuged out of the lysate, and C/EBPβ antibodies or preimmune IgG was added to the precleared lysate, which was then incubated overnight at 4°C with gentle rocking. The next day, protein agarose beads were added into the supernatant and incubated at 4°C for an additional 1 hour. Immunoprecipitated complexes attached to the protein A agarose beads were collected, and reverse‐crosslink of the DNA fragments was achieved by heating the samples at 65°C for 6 hours with agitation. The DNA was subsequently purified using the phenol:chloroform method and precipitated with ethanol. Re‐ChIP was performed using antibodies against coactivator p300 or preimmune IgG. Enrichment of the DNA fragment containing either the putative C/EBP binding site (−515 to −318 bp) on the iNOS promoter or a control sequence (−1796 to −1604 bp) was evaluated by RT‐qPCR using primers summarized in Table 1, and the results were normalized to input.

### Gelatin Zymography

Fresh aortic tissues were ground with a homogenizer in PBS (pH 7.4) containing 0.1% SDS, 0.5% Triton X‐100, and 1 mmol/L phenylmethanesulfonylfluoride. The homogenate was centrifuged at 12 000*g* for 20 minutes at 4°C, and the supernatant was collected and frozen for further analysis. The protein concentration of each sample was determined by Bradford assay using BSA as a standard. Beta‐tubulin was used to confirm equal loading of samples. One volume of sample was then dissolved with 2 volumes of sample buffer, which contained 62.5 mmol/L Tris‐HCl (pH 6.8), 25% glycerol, 4% SDS, and 0.01% bromophenol blue. The samples were subsequently resolved on a 10% SDS‐polyacrylamide gel containing 0.1% (*w*/*v*) gelatin. After electrophoresis, SDS was removed from the gel by washing with 100 mL of renaturation buffer containing 2.5% (*v*/*v*) Triton X‐100 for 1 hour at room temperature with agitation, followed by incubation in 100 mL of development buffer (pH 7.5) containing 50 mmol/L Tris base, 200 mmol/L NaCl, 5 mmol/L CaCl_2_, and 0.02% Brij‐35 for 72 hours at 37°C. The gel was stained with 0.05% Coomassie blue, and gelatinolytic activity was detected as clear bands. The relative molecular weights of proteases were determined by the relation of log Mr (logarithm of molecular mass) to the relative mobility of PageRuler Prestained Protein Ladder (Fermentas).

### Monocyte/Macrophage Depletion

PBS‐ and clodronate‐liposomes were prepared as previously described^22^ using l‐α‐phosphatidylcholine (#P3556; Sigma‐Aldrich), cholesterol (#C75209; Sigma‐Aldrich), and dichloromethylenediphosphonic acid disodium salt (#D4434; Sigma‐Aldrich). Animals received 150 μL intravenous injections of clodronate‐ or PBS‐liposomes every 3 days for 27 days. This dosage has previously been shown to lead to a profound reduction of 80% in circulating monocytes and near disappearance of spleen monocytes with no effect on blood neutrophil count.^23^

### Immunohistochemical Staining

Fresh spleen tissues were processed, embedded, sectioned, and fixed as mentioned above. Immunohistochemical staining of spleen tissues for F4/80^+^‐macrophages was performed using a VectaStain Elite ABC Kit (#PK‐6100; Vecta Laboratories) and a DAB Peroxidase Substrate Kit, 3,3′‐diaminobenzidine (#SK‐4100; Vecta Laboratories) as per the manufacturer's protocols. Briefly, tissue sections were incubated in 3% H_2_O_2_/methanol for 30 minutes at room temperature to eliminate the activity of endogenous peroxidase. Sections were subsequently blocked with 15% NGS for 1 hour at room temperature, followed by incubation with rat anti‐mouse F4/80 antibody and biotinylated secondary antibody for 1 hour at room temperature. Next, sections were incubated with ABC working solution for 30 minutes at room temperature and washed 3 times thereafter. Last, sections were incubated in DAB peroxidase substrate solution until the desired stain intensity developed and counterstained with hematoxylin.

### *Ex Vivo* NO Secretion Assay

The aortas were extracted and rinsed with ice‐cold Krebs–Henseleit buffer via intraventricular injection as described above. The whole aortas were excised, cleaned of periaortic fats, cut into 4‐mm segments, and denuded of endothelial cells by mechanical disruption of the endothelium using a 40‐μm stainless steel wire (Danish Myo Technology). The aortic rings were then transferred to a 96‐well plate and covered with 0.2 mL of phenol red–free DMEM (Life Technologies) containing 2 mmol/L l‐glutamine (Life Technologies), 0.1% BSA, 1× penicillin and streptomycin, and, where appropriate, 10 ng/mL of IL‐1β (PeproTech), 10 ng/mL active TGF‐β1 (PeproTech), 500 μmol/L dexamethasone, 500 μmol/L aspirin (Bayer), or 500 μmol/L aminoguanidine (Tokyo Chemicals Corporation). The plate was incubated for 24 hours at 37°C in 95% humidity and 5% CO_2_. After incubation, 1 μL of the medium was diluted in 200 μL of PBS containing 10 μmol/L DAF‐FM diacetate, followed by incubation at room temperature for 5 minutes in darkness. The fluorescence generated by the interaction of NO with DAF‐FM was subsequently detected using GloMax 20/20 Luminometer (Promega) equipped with a blue fluorescence module and subtracted by the blank (PBS alone with 10 μmol/L DAF‐FM diacetate). The aortic rings that were matched for the anatomical locations were used for the same treatments and compared between WT and S3KO mice.

### Aminoguanidine Treatment

A stock of aminoguanidine solution (80 mg/mL) was prepared by dissolving 4 g of powder in 50 mL of 0.9% saline. After AngII‐filled osmotic pumps were implanted as described above, aminoguanidine was administered intraperitoneally in the high‐dose AngII‐infused mice on the same day in 0.2 mL of 0.9% saline at a dose of 500 mg/kg per day for 28 days.^24^ This dosage has previously been shown to limit aneurysm expansion in elastase‐induced aortic aneurysms despite the presence of arterial hypertension.^25^ For placebo treatment, 0.2 mL of 0.9% saline was injected into the mice instead.

### Statistical Analysis

Values are expressed as mean±standard error of mean (SEM). Statistical tests including the 2‐tailed Mann–Whitney *U*, Friedman's 2‐way nonparametric ANOVA, chi‐square (for aneurysm severity), log‐rank (for survival curves), and Fisher's exact (for aneurysm incidence) tests were performed using SPSS (IBM) and GraphPad Prism (version 4.00) software. *P<*0.05 was considered statistically significant.

## Results

### AngII‐Induced Vascular Inflammation But Not Hypertension Triggers Aortic Aneurysm in S3KO Mice

**Figure 1.**
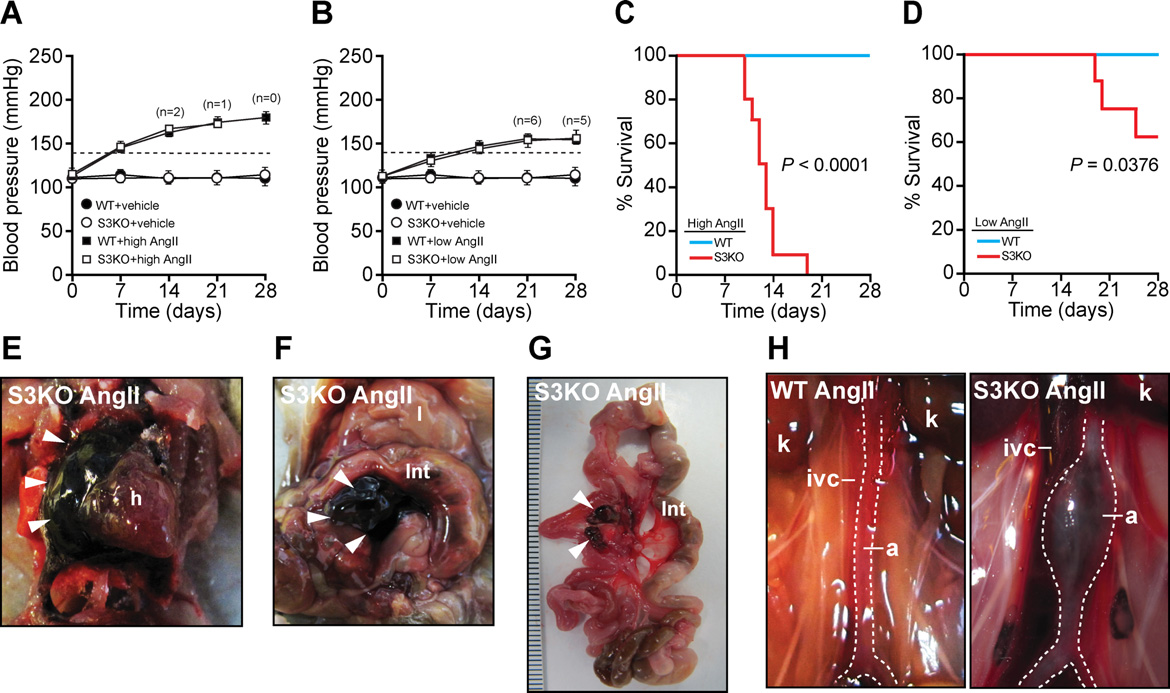
Severe hemorrhage in AngII‐infused S3KO mice. A and B, Blood pressure measurements in high‐dose (1000 ng/kg per minute; n=10 per group) (A) and low‐dose (500 ng/kg per minute; n=8 per group) (B) AngII‐infused WT and S3KO mice. Numbers in brackets indicate the remaining number of S3KO mice at the respective times. Dotted lines indicate the threshold for hypertension, which is clinically defined as an increment of ≥50% over the original blood pressure. Values represent mean±SEM. C and D, Kaplan–Meier plot demonstrating the survival rate after 28 days of high‐dose (C) or low‐dose (D) AngII infusion. *P* values on the survival curves were generated using log‐rank test after comparing with the respective WT counterparts. E through H, Photographs of necropsy findings in S3KO mice after AngII infusion showing hemothorax (E), hemoabdomen (F), mesenteric artery rupture (G), and fusiform infrarenal aortic aneurysm (H). Arrowheads indicate blood clots. Dotted lines demarcate infrarenal aorta from inferior vena cava. Each division on the ruler represents 1 mm. WT, wild type; S3KO, *SMAD3* knockout; AngII, angiotensin II; h, heart; l, liver; int, intestine; k, kidney; ivc, inferior vena cava; a, aorta;.

**Figure 2.**
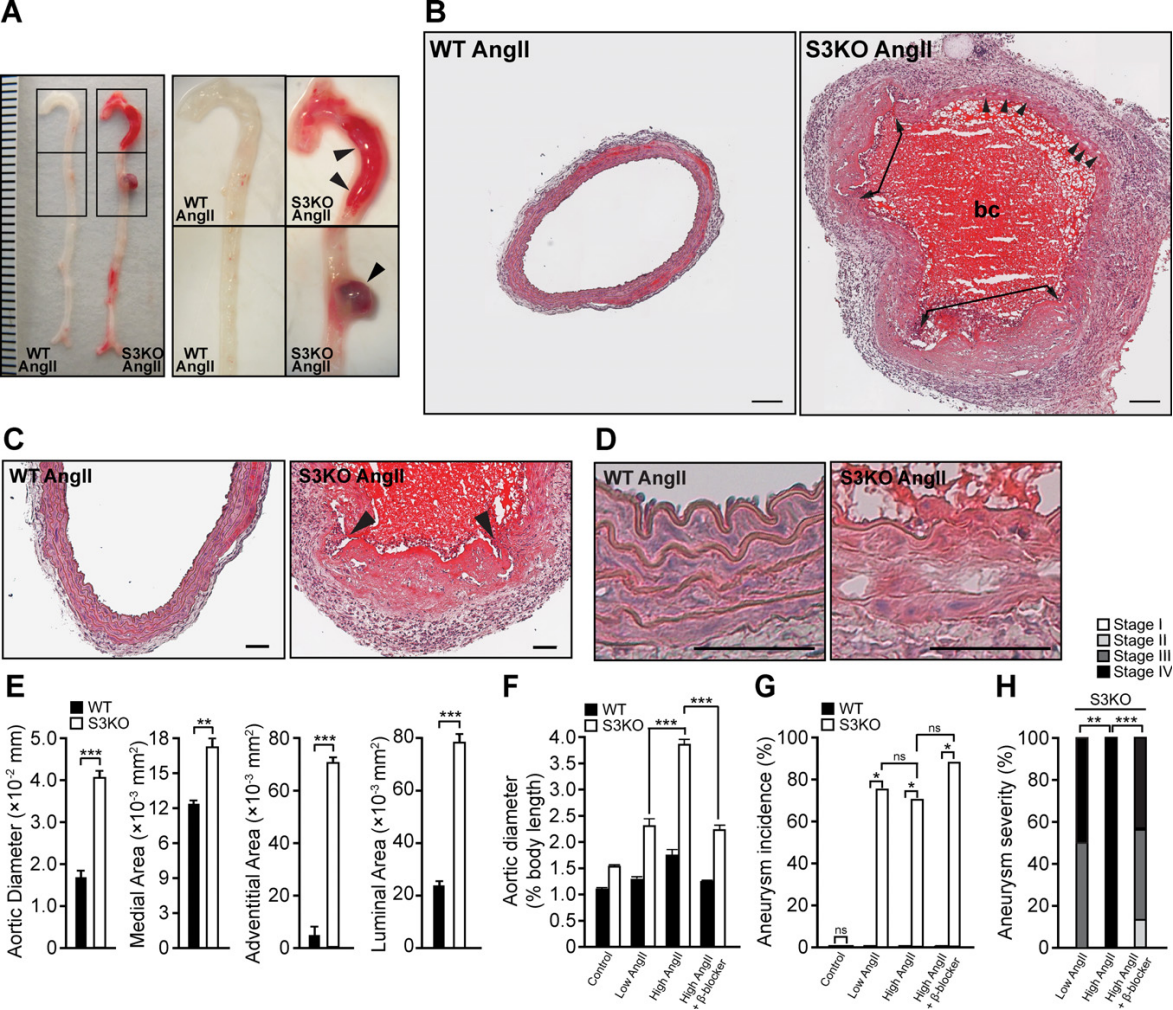
Aortic aneurysm formation in AngII‐infused S3KO mice. A, Gross phenotype of WT and S3KO aorta. Boxed regions on the left panels were magnified and are shown on the right panels. Arrowheads indicate aortic aneurysms. Each division on the ruler represents 1 mm. B, Hematoxylin and eosin staining of WT and aneurysmal S3KO aorta after AngII infusion. Representative image of the aorta was captured at the same anatomical location in both groups. Arrowheads indicate dissection tears within the medial layer. Double‐arrowed connectors indicate the regions where a complete breakage of elastin lamella occurs. Scale bars=200 μm. C and D, Hematoxylin and eosin staining showing disruption of elastic lamella (arrowheads) (C) and medial dissection (D) in S3KO aneurysmal aorta. Scale bars=100 μm. E, Morphometric analysis of the aorta (n=5 per group). F through H, Changes in aortic diameter (F), aneurysm incidence (G), and severity (H) after low‐dose (n=8 per group) and high‐dose (n=10 per group) AngII and β‐blocker (n=8 per group) treatments. Note that differences in aortic diameter between WT and S3KO were significant (*P*<0.001) for control and treatment groups but are not indicated. Values represent mean±SEM. **P<0.01, ***P<0.001. AngII indicates angiotensin II; WT, wild type; S3KO, *SMAD3* knockout; bc, blood clot; ns, not significant.

To examine the effects of AngII, mice were infused with either a high or low dose of AngII for 28 days. High‐dose AngII (1 μg/kg per minutes) has been routinely used for AngII‐induced hypertension.^23^ AngII resulted in hypertension in both S3KO and WT mice after 7 to 10 days of treatment (Figure 1A and 1B). Blood pressure did not vary significantly between the 2 groups. All high‐dose (10 of 10 mice) and ≈37% (3 of 8) of the low‐dose AngII‐infused S3KO mice died within 20 days (Figure 1C and 1D). All WT and vehicle‐infused mice survived the course of treatment. Necropsy examination of S3KO mice revealed hemothorax and hemoabdomen in all cases (Table 2), indicative of thoracic and abdominal aortic rupture (Figure 1E and 1F). Aortic aneurysms/dissections and mesenteric artery rupture occurred in 70% and 30% of the high‐dose AngII‐infused S3KO mice, respectively (Figure 1G and 1H). Aneurysms were not detected in any WT and vehicle‐infused mice. Histologic analysis showed complete disruption of the tunica media at localized regions and widespread medial dissections in the S3KO aneurysmal aorta (Figure 2A through 2D). These findings correlated with the morphometric analysis showing significant aortic dilatation, medial and adventitial thickening, and luminal enlargement in aneurysmal S3KO aortas when compared with their WT counterparts (Figure 2E). Compared with high‐dose AngII, low‐dose AngII treatment in S3KO mice reduced aortic expansion (*P*<0.001) and aneurysm severity (*P*<0.01) and improved the survival rate (*P*=0.0376), but it did not significantly decrease the incidence of aneurysms (Figures 1D, 2F through 2H).

**Table 2.** Necropsy Findings in S3KO Mice Infused with High‐Dose AngII

Features	Percentage
Hemorrhage	
Hemothorax/hemoabdomen	100
Mesenteric artery rupture	30
Aortic aneurysm/dissection	70
Anatomical location of aortic aneurysm/dissection	
Thoracic aorta	
Aortic root	0
Aortic arch	14.3
Descending thoracic aorta	28.6
Abdominal aorta	
Suprarenal aorta	0
Infrarenal aorta	57.1
Type of aneurysm	
Fusiform	57.1
Saccular	43.9

S3KO indicates *SMAD3* knockout; AngII, angiotensin II receptor.

**Figure 3.**
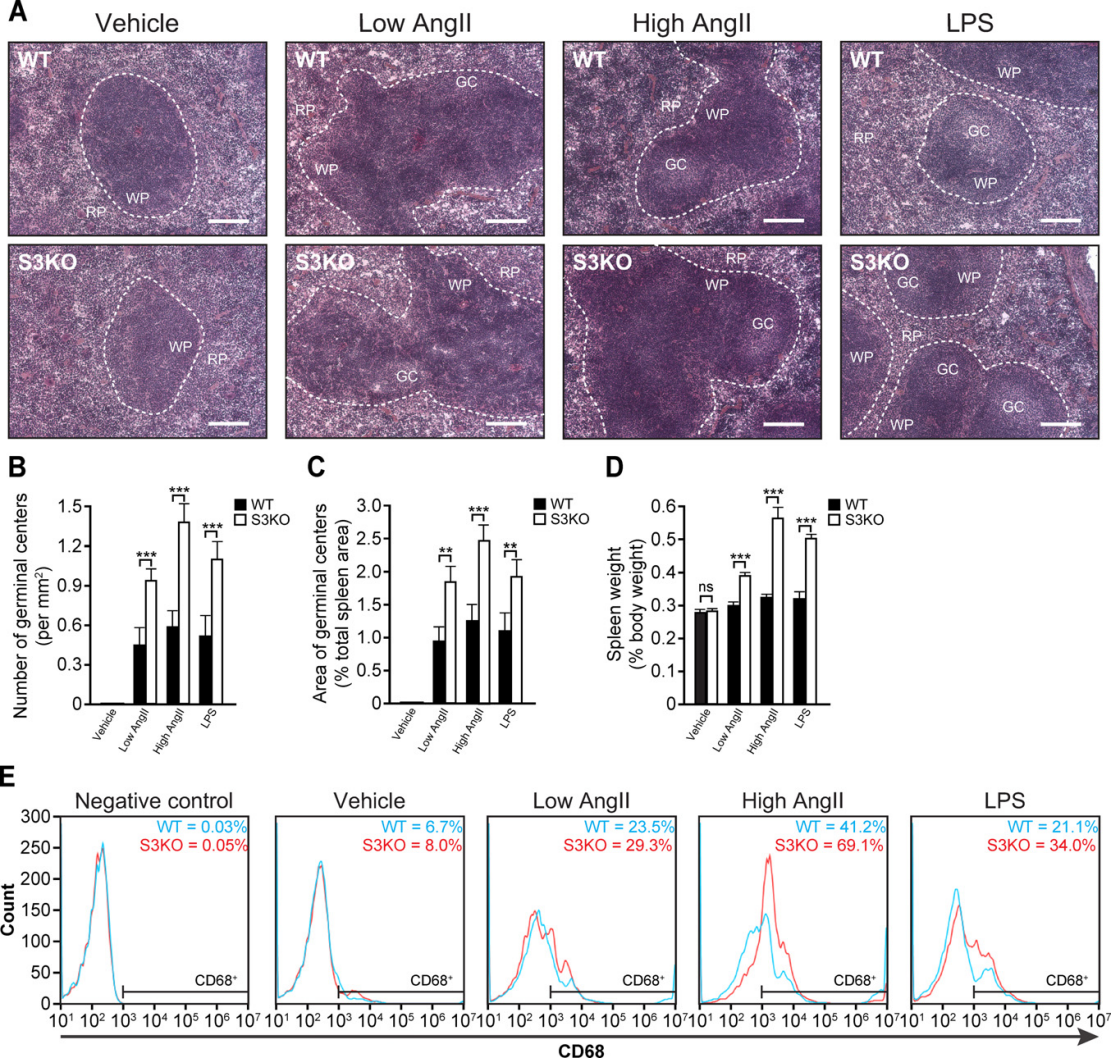
Augmented systemic inflammation induced by AngII or LPS in S3KO mice. A, Hematoxylin and eosin staining of WT and S3KO spleens after AngII or LPS infusion. The presence of systemic inflammation is indicated by the existence of germinal centers (light‐staining region) within the white pulps. Germinal centers are sites where mature B lymphocytes undergo proliferation, differentiation, and somatic hypermutation in response to infection. Note the presence of less active follicles (ie, white pulps) in which germinal center architecture and polarity are less clearly defined in the spleens of low‐dose AngII‐infused mice, indicative of less severe systemic inflammation. Dotted lines demarcate white pulps from red pulps. Scale bars=50 μm. B, Number of germinal centers per square millimeter of spleen section. C, Total area of germinal centers expressed as percentage of total spleen area. D, Spleen weight expressed as percentage of body weight. E, Representative histograms generated from flow cytometry showing the percentage of circulating CD68^+^ monocytes in total white blood cells. Whole blood was lysed with red blood cell lysis buffer, and 10 000 events were recorded. Values represent mean±SEM. **P<0.01, ***P<0.001. AngII indicates angiotensin II; LPS, lipopolysaccharide; S3KO, *SMAD3* knockout; WT, wild type; WP, white pulp; RP, red pulp; GC, germinal center; ns, not significant.

**Figure 4.**
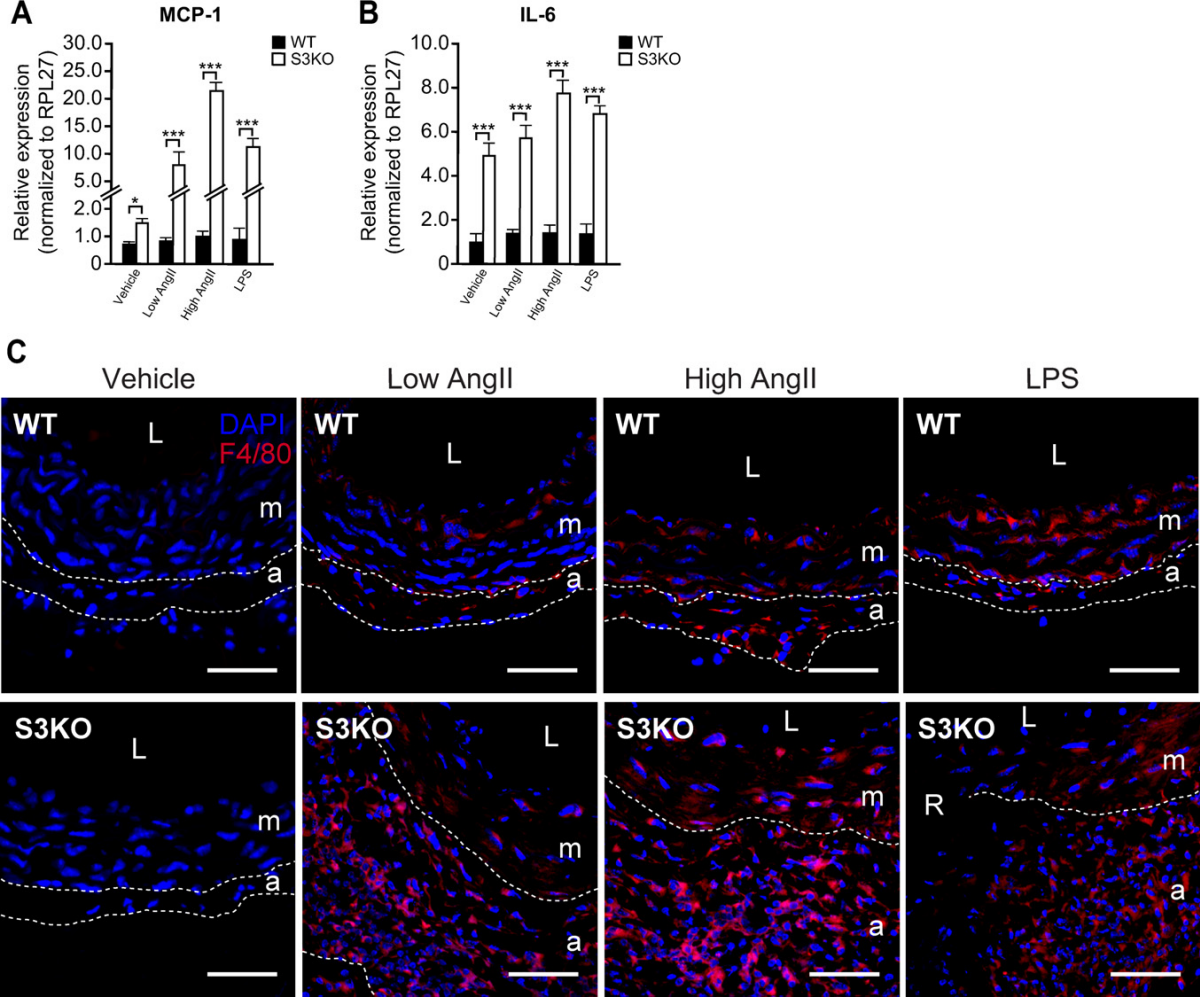
Enhanced vascular inflammation induced by AngII or LPS in S3KO mice. A and B, Relative mRNA expression of proinflammatory cytokines MCP‐1 (A) and IL‐6 (B) in aortas of AngII‐ and LPS‐infused mice (n=3 per group; triplicate experiments were performed). Gene expression was normalized to the expression of RPL27. Values represent mean±SEM. *P<0.05, ***P<0.001. C, Immunofluorescence staining for mature macrophages (F4/80^+^ cells) in S3KO and WT aortas. Note that macrophages were barely detected in aortas of vehicle‐infused mice and that AngII or LPS infusion led to enhanced transmural macrophage infiltration in S3KO aortas compared with their WT counterparts. Dotted lines demarcate adventitial layer from medial layer. Scale bars=50 μm. LPS indicates lipopolysaccharide; S3KO, *SMAD3* knockout; WT, wild type; MCP‐1, monocyte chemotactic protein‐1; IL‐6, interleukin‐6; RPL27, ribosomal protein L27; AngII, angiotensin II; L, lumen; R, rupture; m, tunica media; a, tunica adventitia; ns, not significant.

**Figure 5.**
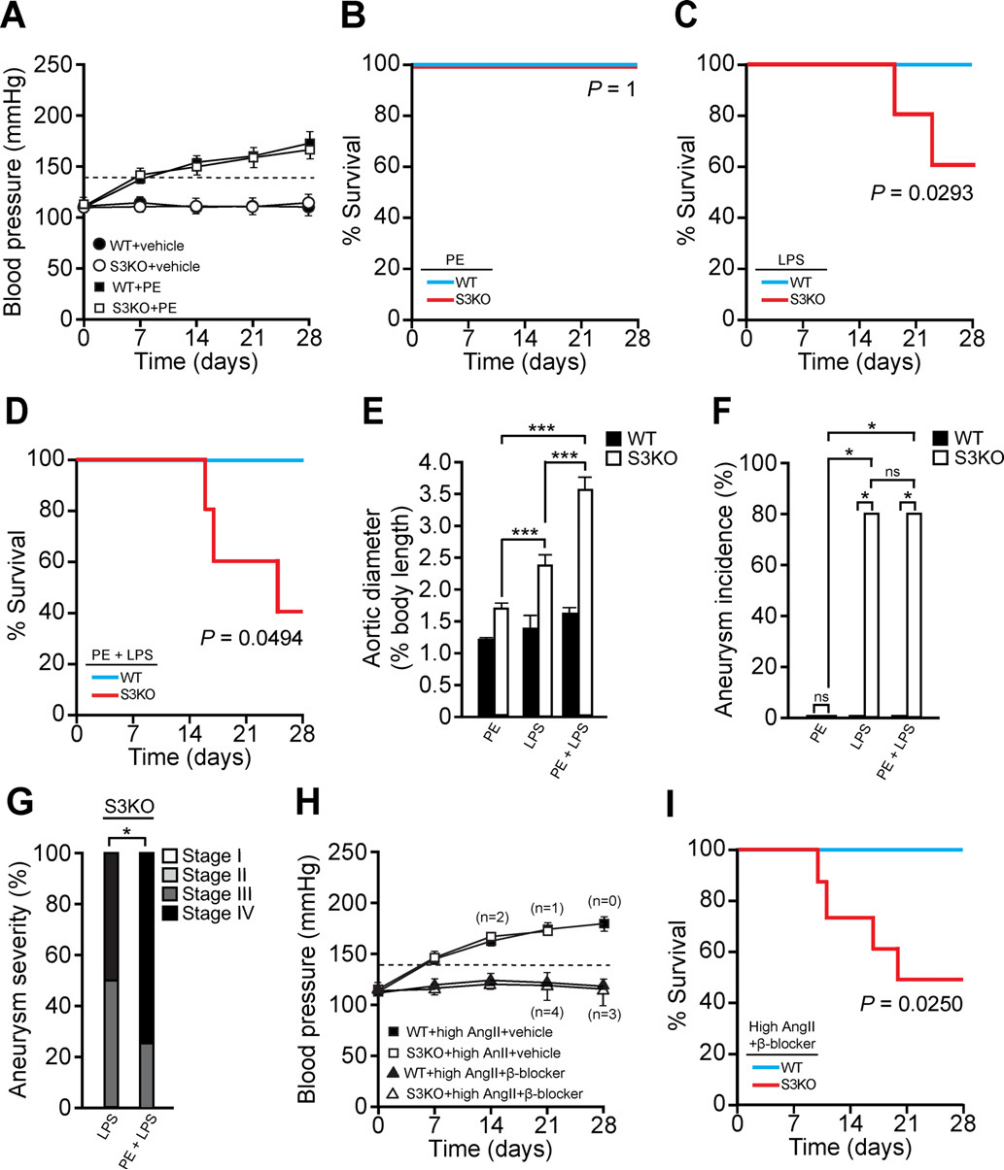
Etiopathology of AngII‐induced aortic aneurysm in S3KO mice. A, Blood pressure measurements in vehicle‐ and PE‐infused WT and S3KO mice. Dotted lines indicate the threshold for hypertension. B through D, Kaplan–Meier plots demonstrating survival rate after 28 days of PE (n=10 per group) (B), LPS (n=10 per group) (C), or concurrent PE and LPS (n=10 per group) (D) infusion. *P* values on the survival curves were generated using the log‐rank test after comparing with the respective WT counterparts. E through G, Changes in aortic diameter (E), aneurysm incidence (F), and severity (G) after PE and/or LPS treatments. Note that differences in aortic diameter between WT and S3KO were significant (*P*<0.001) for all treatments but are not indicated. H, Blood pressure measurements in vehicle‐ and β‐blocker‐treated AngII‐infused WT and S3KO mice. Numbers in brackets indicate the remaining number of S3KO mice at the respective times. I, Kaplan–Meier plot demonstrating the survival rate after 28 days of β‐blocker (n=8 per group) in high‐dose AngII‐infused mice. Values represent mean±SEM. *P<0.05, ***P<0.001. PE indicates phenylephrine; WT, wild type; S3KO, *SMAD3* knockout; LPS, lipopolysaccharide; AngII, angiotensin II; ns, not significant.

AngII has been shown to induce 2 major changes in vascular biology—hypertension and vascular inflammation.^26^ To determine which of these effects caused aortic aneurysms in S3KO mice, we investigated the consequences of AngII‐independent hypertension and vascular inflammation induced by PE and LPS, respectively. The systemic inflammation induced by AngII or LPS was confirmed by spleen enlargement, increased number and area of germinal centers in the spleen, and augmented number of circulating monocytes (Figure 3A through 3E). In contrast, vascular inflammation was indicated by the enhanced aortic expression of proinflammatory cytokines (eg, monocyte chemotactic protein‐1 [MCP‐1] and interleukin [IL]‐6) and macrophage infiltration (Figure 4A through 4C). Interestingly, both the AngII and LPS treatments elicited more severe systemic and vascular inflammation in S3KO mice than in their WT counterparts. Comparable to the vasoconstrictive effects of high‐dose AngII, PE induced similar increases in blood pressure in both S3KO and WT mice (Figure 5A).

**Figure 6.**
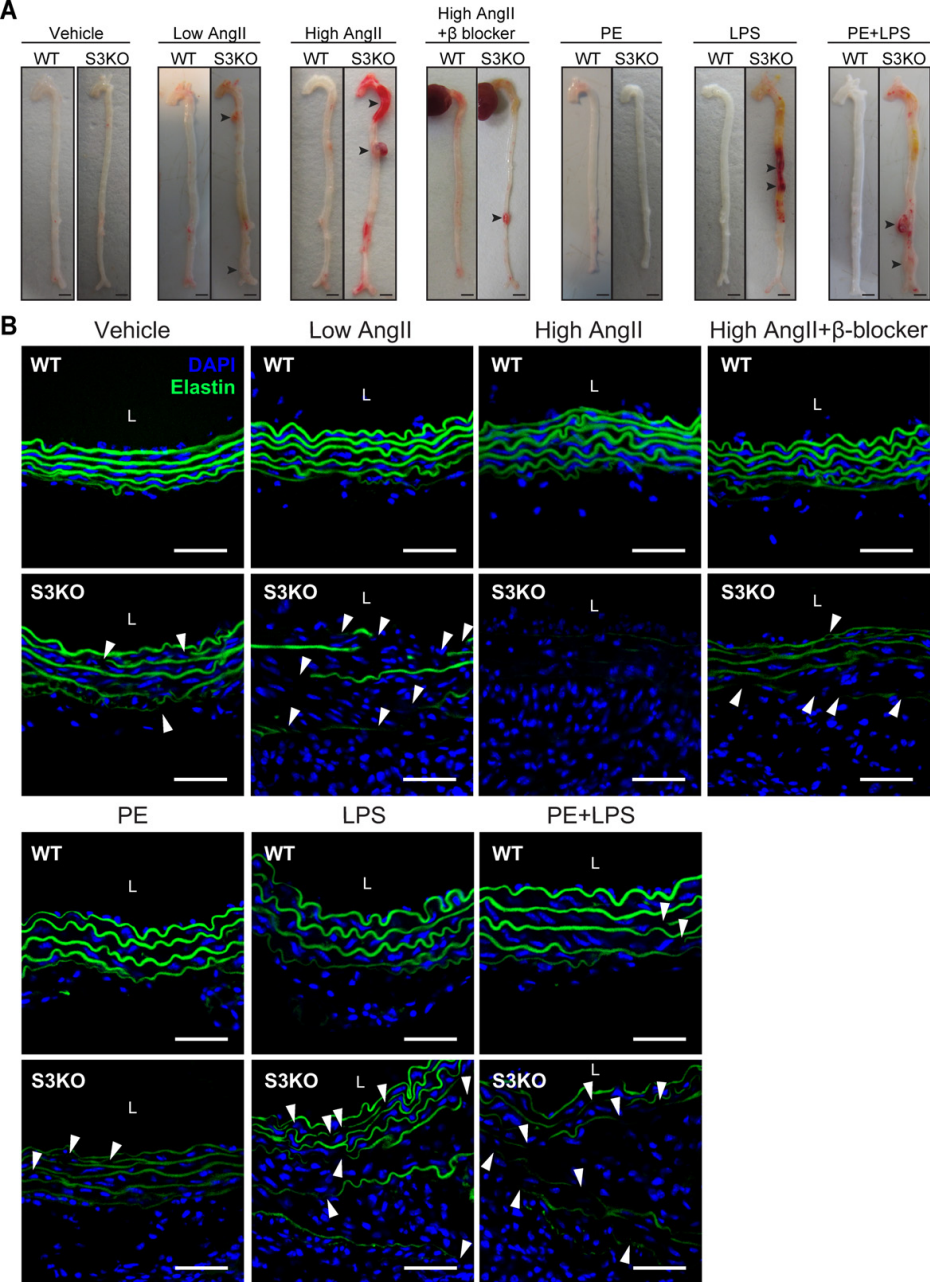
Macroscopic and microscopic morphology of S3KO and WT aortas after drug treatments. A, Representative photographs showing the gross phenotype of WT and S3KO aortas after low‐ and high‐dose AngII, β‐blocker, PE, LPS, and PE+LPS treatments. Arrowheads indicate aortic aneurysms/dissections. Scale bars=2 mm. B, Immunofluorescence staining for elastin in the aorta. Arrowheads indicate elastic fiber fragmentations. Note the complete absence of elastic fibers in high‐dose AngII‐infused S3KO aortas. Aortic dissection is characterized by distant separation of adjacent elastic fibers with the space between 2 fibers occupied by multiple layers of cells, in contrast to monolayer of cells in normal aorta. Scale bars=50 μm. S3KO indicates *SMAD3* knockout; WT, wild type; AngII, angiotensin II; PE, phenylephrine; LPS, lipopolysaccharide; L, lumen.

We noted that PE‐induced hypertension alone did not trigger aortic aneurysms in S3KO mice, and all PE‐treated S3KO and WT mice survived the course of treatment (Figure 5B), whereas LPS‐induced inflammation caused aortic aneurysms and death in 80% (8 of 10) and 40% (4 of 10) of S3KO mice, respectively. The resulting rates of aortic expansion, aneurysm incidence and severity, and mortality rate were comparable to those of the low‐dose AngII‐infused S3KO mice (Figure 5C, 5E through 5G, compare with Figures 1D, 2F through 2H). Notably, compared with LPS treatment alone, concurrent treatment of PE and LPS led to a more severe aneurysm phenotype and a higher death rate of 60% (6 of 10) in S3KO mice, but the incidence of aneurysm remained unchanged (Figure 5D through 5G). Furthermore, the administration of a blood pressure–lowering β‐blocker in the high‐dose AngII‐infused S3KO mice maintained normotension in both S3KO and WT mice throughout the course of AngII treatment (Figure 5H). When compared with the AngII‐infused group, S3KO mice receiving β‐blocker treatment had significantly reduced aortic expansion (Figure 2F), aneurysm severity (Figure 2H), and death rate (5 of 8 mice died) (Figure 5I) but failed to significantly lower aneurysm incidence (Figure 2G). The vascular pathology was clearly reflected by the gross morphology of the aortas, as well as the presence of fragmented elastic fibers and aortic dissections (Figure 6A and 6B). Together, these findings suggest that hypertension merely acts as a risk factor, whereas vascular inflammation initiates aortic aneurysm in S3KO mice.

### S3KO Aortas Display Pathological Features of a Connective Tissue Disorder

**Figure 7.**
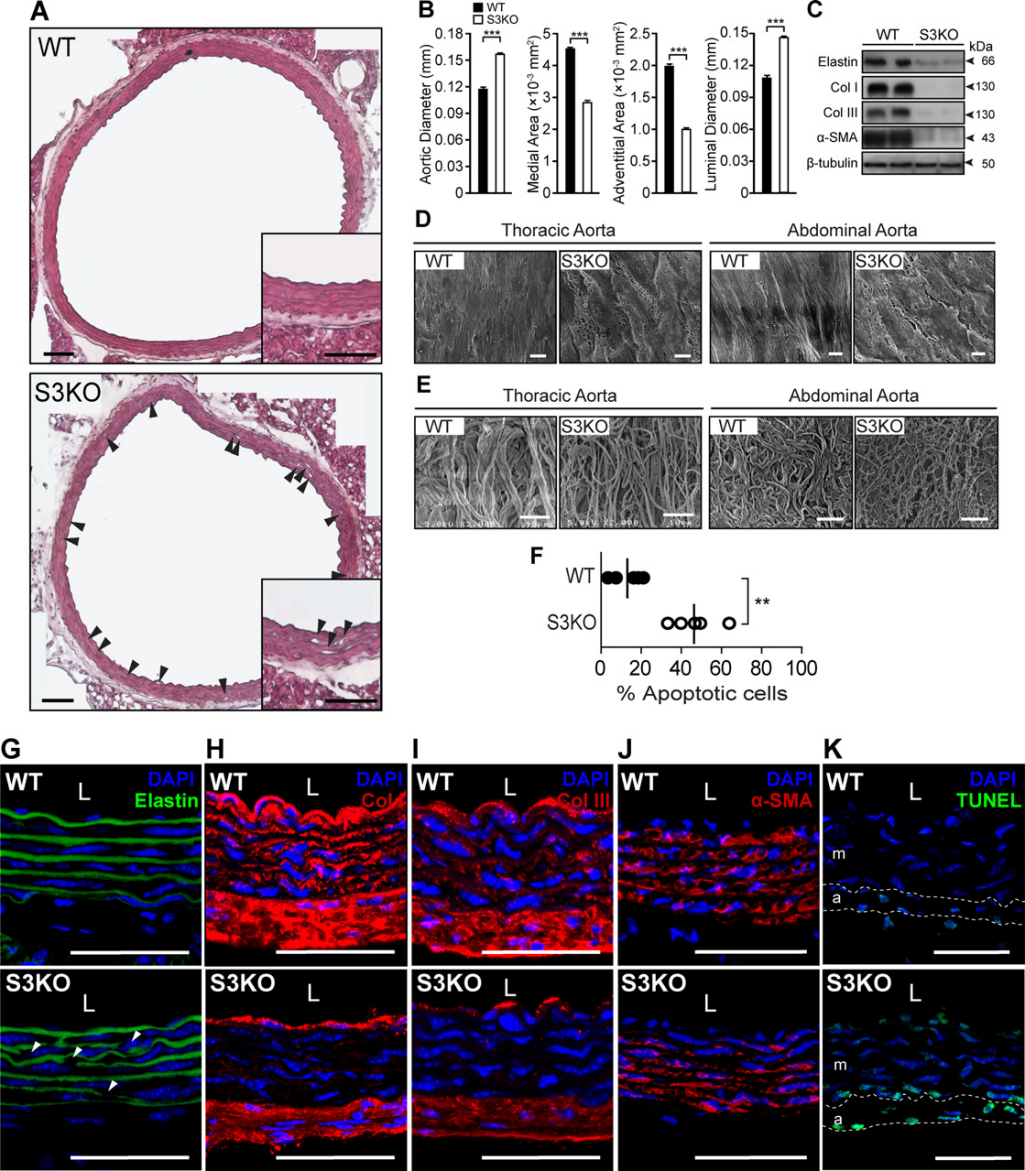
Phenotypes of S3KO and WT aortas before AngII infusion. A, Hematoxylin and eosin staining of the aorta showing numerous dissection tears (arrowheads) within the medial layer of S3KO aorta. Inset showing magnified image of a representative region. Scale bars=100 μm. B, Morphometric analyses of the aorta (n=8 per group). C, Western blot analysis showing the expression of extracellular matrix components (ie, elastin, type I collagen, and type III collagen) and VSMC marker (α‐SMA) in S3KO and WT aortas. Beta‐tubulin served as a loading control. D and E, Scanning electron microscopy of internal elastic lamellae (D) and adventitial collagen fibers (E) of thoracic and abdominal aortas (n=3 per group) showing enlarged fenestrations in the elastic lamella and thinner collagen fibers in S3KO mice, compared with WT counterparts. Note the disorganization of the elastic lamella with small gullies and ridges and the dark hollow area underneath the collagen meshwork in S3KO aortas. Scale bars=10 μm. F, Percentage of apoptotic cells present in the aortic section (n=5 per group). It was calculated by the number of total TUNEL‐positive cells over the number of total DAPI‐Dpositive cells. Note WT=13.93±3.38% vs S3KO=47.21±5.15%. G through K, Immunofluorescence staining for elastin (G), type I collagen (H), and type III collagen (I), α‐SMA (J), and apoptotic cells (K) in S3KO and WT aortas. Note the presence of elastin fragmentation (arrowheads), reduced collagen deposition in the medial and adventitial layers, and decreased α‐SMA expression in the S3KO aorta compared with WT counterparts. Note the increased number of apoptotic cells present in the medial layer of S3KO aortas when compared with WT counterparts. Images were taken at the same anatomical locations in both groups. Dotted lines demarcate the adventitial layer from the medial layer. Scale bars=50 μm. Values represent mean±SEM. **P<0.01, ***P<0.001. S3KO indicates *SMAD3* knockout; WT, wild type; TUNEL, terminal deoxynucleotidyl transferase‐mediated dUTP nick end labeling; Col, collagen; α‐SMA, α‐smooth muscle actin; L, lumen; m, tunica media; a, tunica adventitia; VSMC, vascular smooth muscle cell.

To investigate the cause of this severe pathology, we studied the morphologies of S3KO and WT aortas in the absence of AngII. The histology of S3KO aortas revealed medial dissections that recapitulated the characteristic mucoid medial degeneration observed in AOS (Figure 7A).^4^ Furthermore, morphometric analysis revealed aortic dilatation characterized by a significant increase in aortic diameter and luminal enlargement (Figure 7B). In particular, thinning of the medial and adventitial layers suggested the presence of structural matrix degradation. Hence, we next examined the ultrastructure of the aortic extracellular matrix. Scanning electron microscopy showed that both the thoracic and abdominal aortas of S3KO mice displayed disorganization and enlarged fenestrations in the internal elastic lamella and thinner adventitial collagen fibers compared with the WT controls (Figure 7D and 7E). These data suggest that the observed aortic structural defects likely occur throughout the entire aorta rather than being restricted to certain anatomical locations.

We also found that S3KO aortas exhibited elastin fragmentation, reduced collagen deposition in the aortic wall, and decreased expression of α‐SMA, a VSMC marker (Figure 7G through 7J). Western blot analysis verified that these structural protein levels were reduced in S3KO aortas (Figure 7C). Furthermore, a significant increase in the number of apoptotic cells in the S3KO aortic wall was detected, indicating loss of vascular cells (Figure 7F and 7K). These results underscore the presence of pathological hallmarks of a connective tissue disorder observed in aortic aneurysms.^27^

### S3KO Aortas Exhibit Defective Biomechanics and Contractile Function

**Figure 8.**
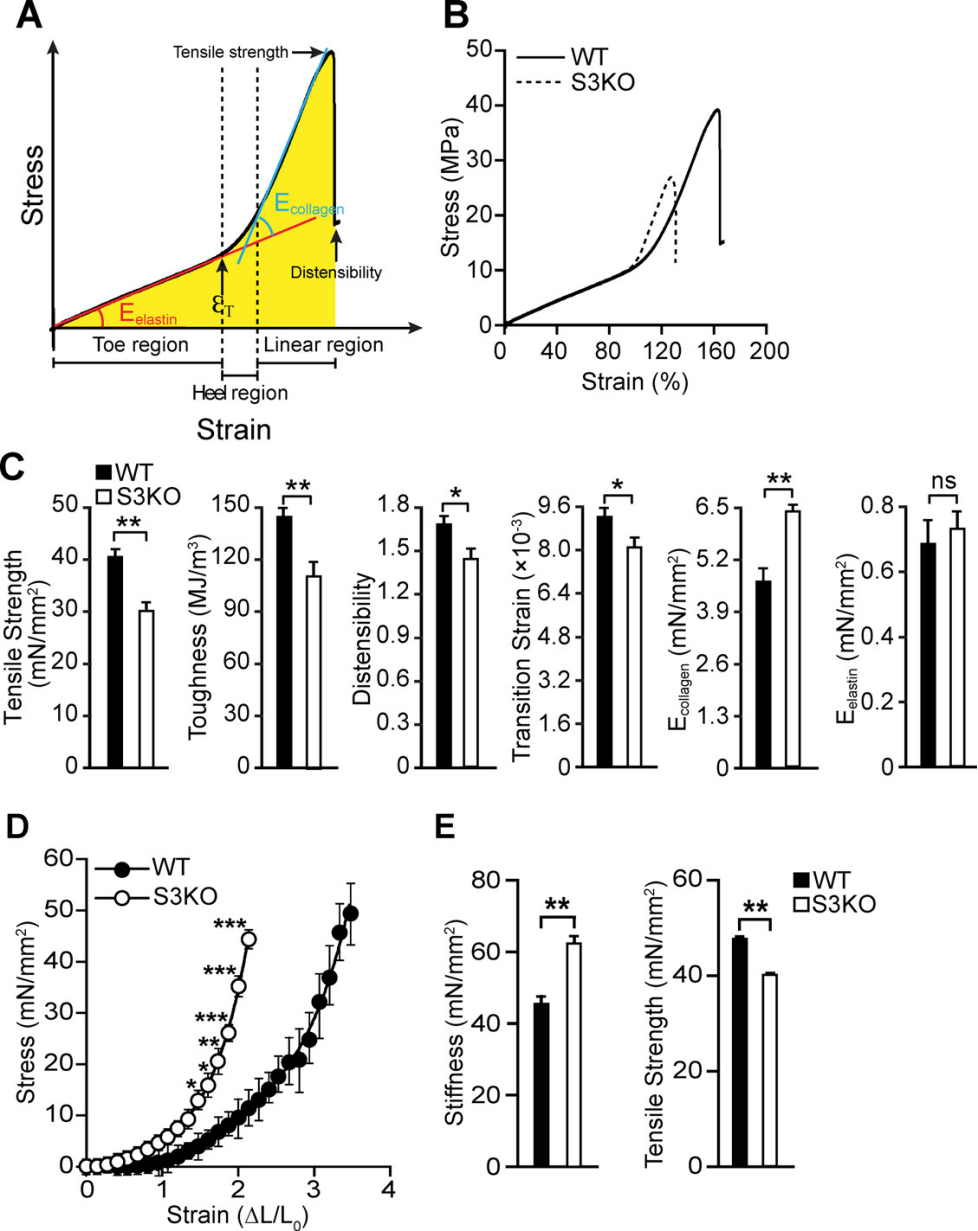
Defective biomechanical properties in S3KO aorta. A, Schematic diagram of a typical stress–strain curve of aorta. E_elastin_ and E_collagen_ denote the maximum tangential moduli from which the stiffness of elastic lamella and collagen fibers are calculated, respectively. Note that the initial linear slope is mainly contributed by elastic lamella. As the stress increases, collagen fibers start to engage to maintain the aortic wall shape, and this creates a heel region on the stress–strain curve. A steeper linear slope after the heel region indicates complete engagement of all the collagen fibers present in the aorta. In aneurysmal aortas, elastin degradation leads to collagen engagement at a lower transition strain (ε_T_) because of compromised distensibility or elasticity. Tensile strength is the maximum stress that an aorta can withstand while being stretched before rupture, whereas distensibility (maximum strain) reflects the elasticity of an aorta. Toughness (also called resistance to rupture) is measured by calculating the area under the curve (yellow shaded area) and is defined as the amount of energy per volume an aorta can absorb before rupturing. B, Representative stress–strain curve of S3KO and WT aortas. C, Biomechanical properties of the aorta during uniaxial tensile tests (n=8 per group). D and E, Biomechanical properties of S3KO and WT aortas in circumferential tensile tests (n=5 per group). D, Stress–strain curve of S3KO and WT aortas generated from circumferential tensile tests. E, Stiffness and tensile strength of the aorta. Values represent mean±SEM. *P<0.05, **P<0.01, ***P<0.001; ns, not significant. S3KO indicates *SMAD3* knockout; WT, wild type.

Given the structural abnormalities observed in S3KO aortas, profound changes in their biomechanical properties and physiological functions would be expected. Therefore, we performed uniaxial (longitudinal) and circumferential tensile tests to verify this correlation. In uniaxial tests, both the untreated S3KO and WT aortas exhibited pronounced “toe‐region” characteristics typical of soft biological tissues, and the former produced a distinctively smaller stress–strain curve than the latter (Figure 8A and 8B). Measurements of the mechanical properties of S3KO aortas revealed significant reductions in tensile strength, toughness, distensibility, and transition strain (Figure 8C). Moreover, the stiffness of collagen fibers was also significantly higher in S3KO aortas compared with WT controls, although the stiffness of the elastic lamella was similar between the 2 groups. Circumferential tensile tests consistently demonstrated that the stiffness of S3KO aortas was significantly higher than that of the WT controls, whereas tensile strength was reduced (Figure 8D and 8E).

**Figure 9.**
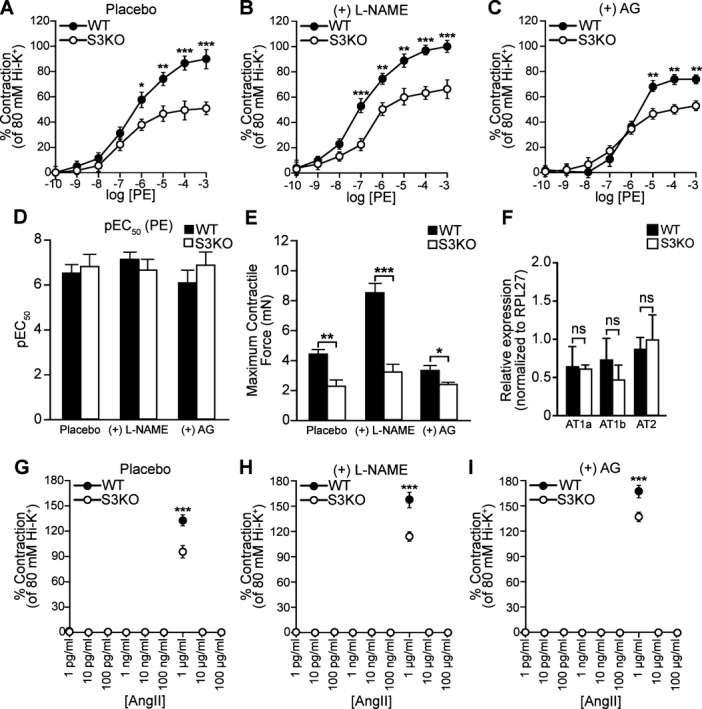
Defective contractile function of S3KO aorta. A through C, PE‐induced contraction of WT and S3KO aortic rings treated with placebo (A), 200 μmol/L l‐NAME (B), or 200 μmol/L aminoguanidine (C) (n=4 per group; duplicate experiments were performed). Contraction was measured at PE concentrations ranging from 10^−10^ to 10^−3^ mol/L, and concentration–response curves were constructed. Note that contraction is expressed as the percentage of maximal contraction induced by incubation of aortic rings in 80 mmol/L high potassium (Hi‐K^+^) buffer. For placebo and l‐NAME treatment, endothelium‐intact aortic segments were used, whereas endothelium‐denuded aortas were used in aminoguanidine treatment to eliminate the vasorelaxant effect of eNOS‐derived NO. D, pEC_50_ of WT and S3KO aortas in response to PE, indicating tissue sensitivity toward the drug. Note that pEC_50_ is the negative logarithm of EC_50_, which refers to the concentration of a drug that induces a half‐maximal response. E, Maximal contraction force generated when incubated with 80 mmol/L Hi‐K^+^ buffer. F, Aortic mRNA level of AngII type 1a (AT1a), type 1b (AT1b), and type 2 (AT2) receptors in WT and S3KO mice (n=3 per group; triplicate experiments were performed). Gene expression was normalized to RPL27. G through I, AngII‐induced contraction in *ex vivo* aortic segments treated with placebo (G), 200 μmol/L l‐NAME (H), or 200 μmol/L aminoguanidine (I) (n=5 per group; duplicate experiments were performed). Note that AngII elicited a rapid and transient contraction of infrarenal aortic segments at a concentration of 1 μg/mL, whereas minimal (<10%) contraction was induced in the thoracic aortic segments of both groups (data not shown). Values represent mean±SEM. *P<0.05, **P<0.01, ***P<0.001. PE indicates phenylephrine; WT, wild type; S3KO, *SMAD3* knockout; l‐NAME, Nω‐nitro‐l‐arginine methyl ester; eNOS, endothelial NO synthase; NO, nitric oxide; AG, aminoguanidine; RPL27, ribosomal protein L27; AT, angiotensin II receptor; AngII, angiotensin II; ns, not significant.

Regarding aortic functionality, we observed a reduced response to PE‐induced contraction in endothelium‐intact aortic segments from S3KO mice compared with their WT counterparts (Figure 9A). To eliminate the influence of endothelial NO synthase (eNOS)–derived NO, we tested the aortic functions in the presence of an eNOS‐selective inhibitor, l‐NAME. A significant decrease of ≈40% in the maximal PE‐induced contraction was observed in S3KO aortas when compared with WT aortas (Figure 9B). Treatment of endothelium‐denuded aortic segments with the iNOS‐selective inhibitor aminoguanidine further showed that the compromised aortic contractility observed in S3KO aortas was independent of the vasorelaxant effects of both iNOS and eNOS as well as of drug sensitivity (Figure 9C and 9D). In addition, the maximum contractile force generated in response to potassium‐induced complete depolarization was also significantly lower in S3KO aortas, demonstrating vasomotor dysfunction in these mice (Figure 9E). Similar findings were also observed for the AngII‐induced contraction, in which the reduced aortic contractility was not caused by differential expression of AngII receptors (Figure 9F through 9I). Taken together, these data indicate that SMAD3 deficiency causes pathologic changes in aortic biomechanics as well as functional deterioration and that these changes parallel the characteristic features of connective tissue disorders in aortic aneurysms.^20,28^

### Elevated iNOS‐Derived NO Production in Vehicle‐ and AngII‐Infused S3KO Aortas

**Figure 10.**
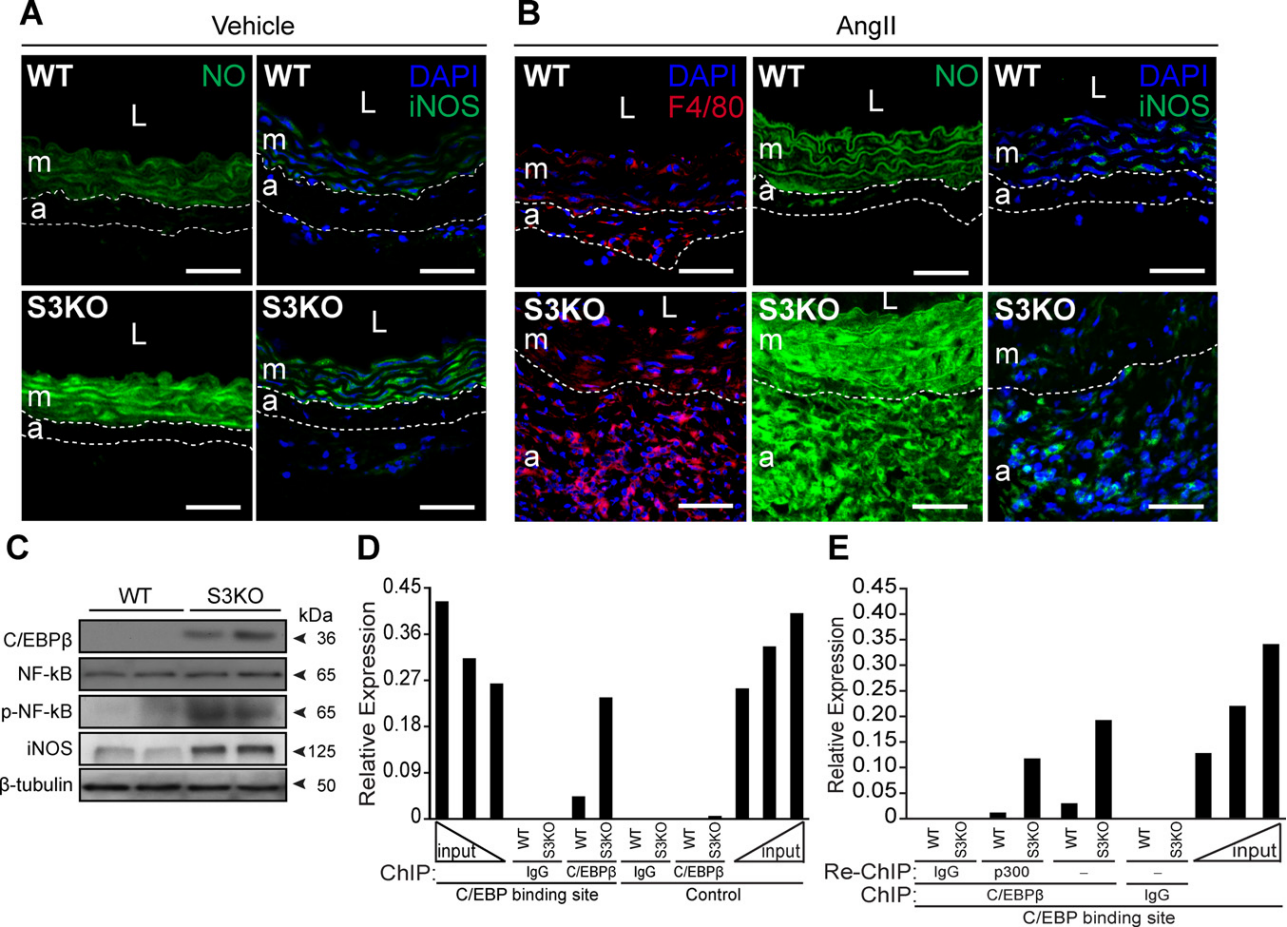
Molecular mechanism by which SMAD3 deficiency causes enhanced iNOS‐derived NO production in aorta. A and B, Fluorescence staining for NO, iNOS, and F4/80 in vehicle‐ (A) and AngII‐infused (B) WT and S3KO aortas. Dotted lines demarcate adventitial layer from medial layer. Scale bars=50 μm. C, Western blots showing increased C/EBPβ, phosphorylated NF‐κB, and iNOS in S3KO aorta. Beta‐tubulin served as a loading control. D, In vivo ChIP of C/EBP binding site within the mouse iNOS promoter and a control region 2 kb upstream of the C/EBP site using antibodies against C/EBPβ or preimmune IgG. The gene fragments in the immunoprecipitated chromatin were quantified by RT‐qPCR. Aliquots of the chromatin were also analyzed before immunoprecipitation (input). E, Re‐ChIP was performed using antibodies against coactivator p300. AngII indicates angiotensin II; iNOS indicates inducible nitric oxide synthase; WT, wild type; NO, nitric oxide; S3KO, *SMAD3* knockout; C/EBP, CCAAT/enhancer binding protein; L, lumen; m, tunica media; a, tunica adventitia; NF‐κB, nuclear factor‐kappaB.

Elevated iNOS expression and NO production have been implicated in rodent and human aortic aneurysms.^29–30^ Indeed, we detected higher NO and iNOS expression in the medial layers of vehicle‐infused S3KO aortas, indicating that VSMCs were primarily involved in the iNOS‐mediated NO production (Figure 10A). Furthermore, AngII infusion caused an increase in the transmural infiltration of macrophages in the aneurysmal S3KO aortic wall (Figure 10B). This observation may partly explain the thickening of the medial and adventitial layers (Figure 2E). These cells also positively stained for NO and iNOS, which is consistent with the findings that macrophage recruitment into aneurysmal walls promotes aneurysm progression by stimulating iNOS expression and subsequent NO production.^30–31^

### Elevation of iNOS Expression in S3KO Aortas Is Indirectly Mediated Via C/EBPβ

In vitro findings have previously indicated that TGF‐β1 inhibits VSMC activation markers such as iNOS and IL‐6 via its downstream effector SMAD3.^32^ Previous studies have also demonstrated that C/EBP and NF‐κB binding sites are critical for cytokine induction of the iNOS promoter in VSMCs.^33–34^ Using Western blot analysis, we found increased expression of C/EBPβ and phosphorylated NF‐κB with a concomitant upregulation of iNOS expression in untreated S3KO aortas (Figure 10C). These results suggest that elevated iNOS expression in S3KO aortas may result from increased transcriptional activity at the C/EBP or NF‐κB binding sites within the iNOS promoter. As an in vitro study showed that SMAD3 regulates the transcriptional activity of the iNOS promoter via C/EBPβ,^32^ we next performed in vivo ChIP using anti‐C/EBPβ antibodies on primary vascular cells isolated from untreated S3KO and WT aortas. ChIP assays revealed that there was an increased association of C/EBPβ with a putative C/EBP binding site spanning −515 to −318 bp within the iNOS promoter in S3KO cells (Figure 10D). A re‐ChIP assay using anti‐p300 antibodies further demonstrated that the DNA–protein association resulted in the recruitment of a coactivator, p300, to this element, thereby verifying the regulatory function of this C/EBP binding element (Figure 10E). No amplification was detected using preimmune IgG or a control sequence (−1796 to −1604 bp). These findings suggest that SMAD3 deficiency indirectly enhances iNOS expression via augmented C/EBPβ expression.

### S3KO Aortas Exhibit Enhanced Elastinolytic MMP‐2 and MMP‐9 Activities

**Figure 11.**
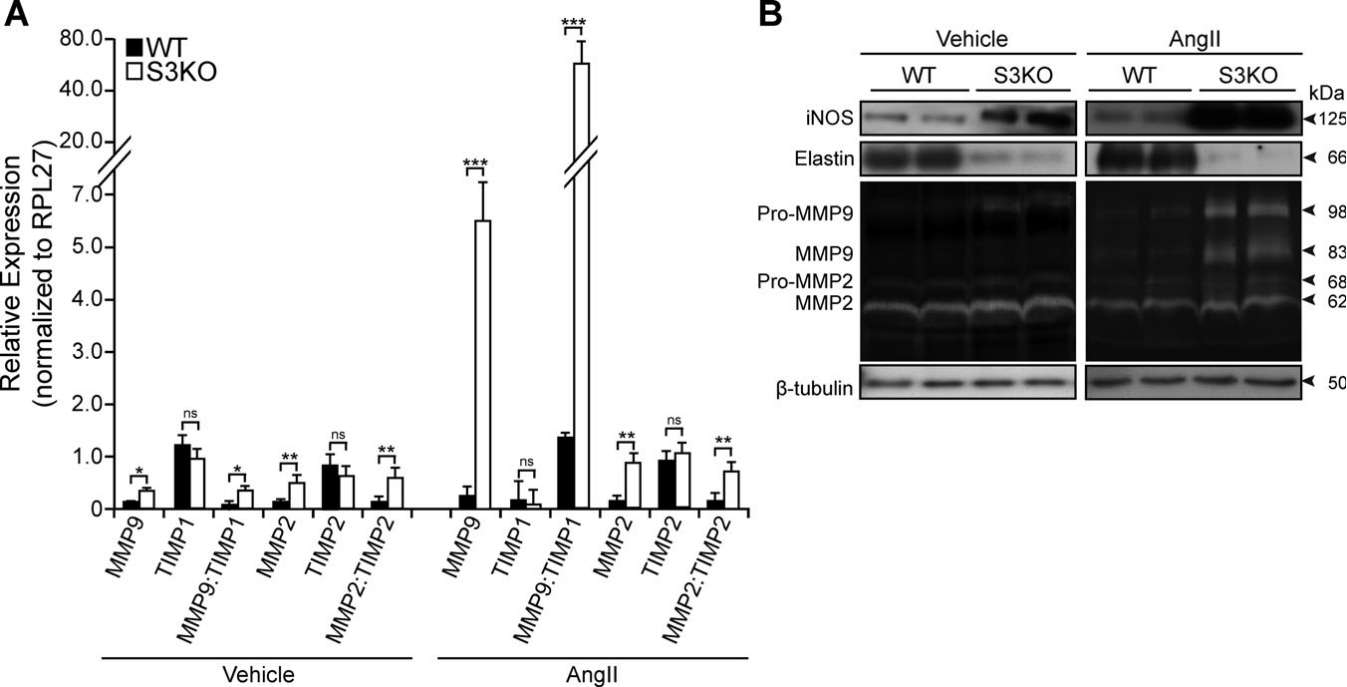
Augmented expression and activity of elastolytic MMP‐2 and MMP‐9 in S3KO aorta. A, Relative mRNA expression of MMP‐2, MMP‐9, and their respective inhibitors in vehicle‐ and AngII‐infused WT and S3KO aortas (n=3 per genotype; triplicate experiments were performed). Gene expression was normalized to RPL27. Values represent mean±SEM. *P<0.05, **P<0.01, ***P<0.001. B, Western blot analysis of iNOS and elastin with gelatin zymography showing enhanced enzymatic activities of MMP‐2 and MMP‐9 in vehicle‐ and AngII‐infused S3KO and WT aortas. Beta‐tubulin served as a loading control. MMP indicates matrix metalloproteinase; WT, wild type; S3KO, *SMAD3* knockout; RPL27, ribosomal protein L27; iNOS, inducible nitric oxide synthase; TIMP, tissue inhibitor of metalloproteinases; AngII, angiotensin II; ns, not significant.

The production of iNOS‐derived NO has previously been shown to increase the expression and activities of elastolytic MMP‐2 and MMP‐9.^35^ Further, the presence of the activated forms of MMP‐2 and MMP‐9 has also been reported in patients with aortic aneurysms and implicated in the degradation of the extracellular matrix.^36^ To assess this possibility, we studied the expression of these enzymes and their respective inhibitors. Significant increases in the expression of MMP‐2 and MMP‐9 were found in both vehicle‐ and AngII‐infused S3KO aortas (Figure 11A). Although the mRNA levels of tissue inhibitor of metalloproteinase‐1 (TIMP‐1) and TIMP‐2 were similar between S3KO and WT aortas, the MMP‐9:TIMP‐1 and MMP‐2:TIMP‐2 ratios were significantly higher in S3KO aortas. These measures were markedly augmented in S3KO mice on AngII infusion, reaching ≥24‐ and ≥46‐fold higher than those of their WT counterparts for MMP‐9 and the MMP‐9:TIMP‐1 ratio, respectively. In contrast, the expression of MMP‐2 and the MMP‐2:TIMP‐2 ratio were modestly increased in S3KO aortas compared with WT aortas. As expected, gelatin zymography detected drastically enhanced enzymatic activities of both pro‐MMP‐9 and active MMP‐9, coincident with moderately enhanced MMP‐2 activity in S3KO aortas (Figure 11B). These data correlated with elevated iNOS expression and reduced elastin content in S3KO aortas, supporting the notion that aortic elastolysis in aneurysmal aortas is partly mediated by iNOS‐derived NO‐activated MMP‐2 and MMP‐9.

### Monocytes/Macrophages Are Critical Mediators of Aortic Aneurysm Formation in AngII‐Infused S3KO Mice

**Figure 12.**
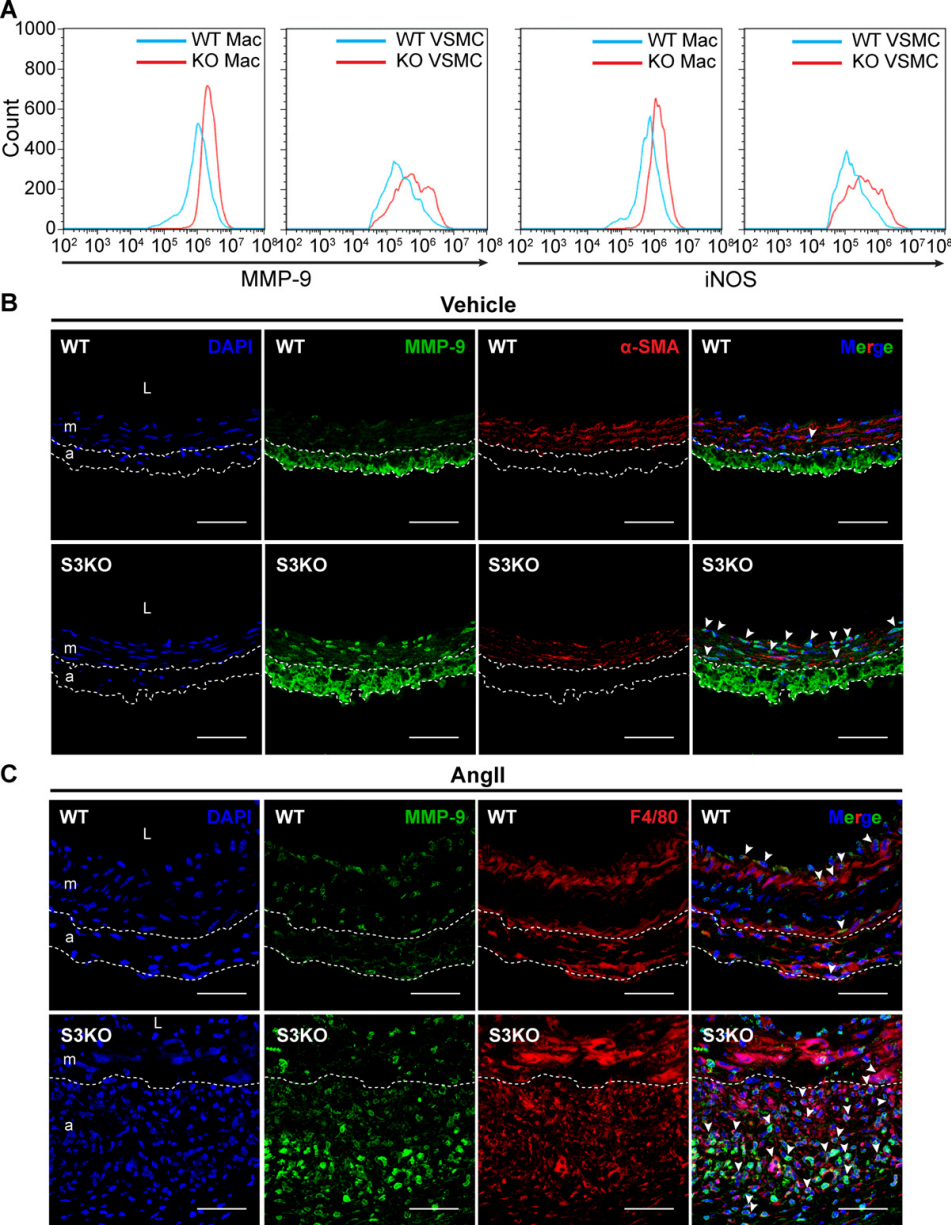
Localization of MMP‐9 in vehicle‐ and AngII‐infused S3KO and WT aortas. A, Flow cytometry analysis of MMP‐9 and iNOS expression in peritoneal monocytes/macrophages and VSMCs of S3KO and WT mice. Histograms showing the expression of MMP‐9 or iNOS in 10 000 events of CD68^+^‐monocytes/macrophages and α‐SMA^+^‐VSMCs. Note that S3KO monocytes/macrophages and VSMCs express higher levels of MMP‐9 and iNOS than their WT counterparts. Importantly, both MMP‐9 and iNOS are expressed at a higher level in monocytes/macrophages than in VSMCs. B and C, Double immunofluorescence staining for MMP‐9 and α‐SMA (B) or F4/80 (C) in vehicle‐ and AngII‐infused aortas, respectively. Dotted lines demarcate adventitial layer from medial layer. Arrowheads indicate colocalization of MMP‐9 with either α‐SMA or F4/80. Scale bars=50 μm. MMP indicates matrix metalloproteinase; S3KO, *SMAD3* knockout; WT, wild type; Mac, macrophage; iNOS, inducible nitric oxide synthase; VSMC, vascular smooth muscle cell; α‐SMA, α‐smooth muscle actin; L, lumen; m, tunica media; a, tunica adventitia.

Our observations of macrophage infiltration within the vessel walls suggested a pathogenic role of this cell subset in disease initiation in S3KO mice. Consistent with this hypothesis, we noted enhanced expression of iNOS and MMP‐9 in both monocytes/macrophages and VSMCs isolated from the untreated S3KO mice compared with their WT counterparts, with higher expression detected in monocytes/macrophages than in VSMCs (Figure 12A). Immunofluorescence staining for MMP‐9 revealed enhanced immunoreactivity in VSMCs within the medial layer of vehicle‐treated S3KO aortas (Figure 12B). However, few or no macrophages were detected in the aortas, suggesting that VSMCs are responsible for the production of MMP‐9 in the initial stage before aneurysm progression (Figure 4C). In contrast, most of the MMP‐9^+^ cells in the AngII‐infused S3KO aortas were in the tunica adventitia; these cells were also positively stained for F4/80, indicating that infiltrated macrophages within the vessel walls are mostly responsible for the production of MMP‐9 during disease progression (Figure 12C).

**Figure 13.**
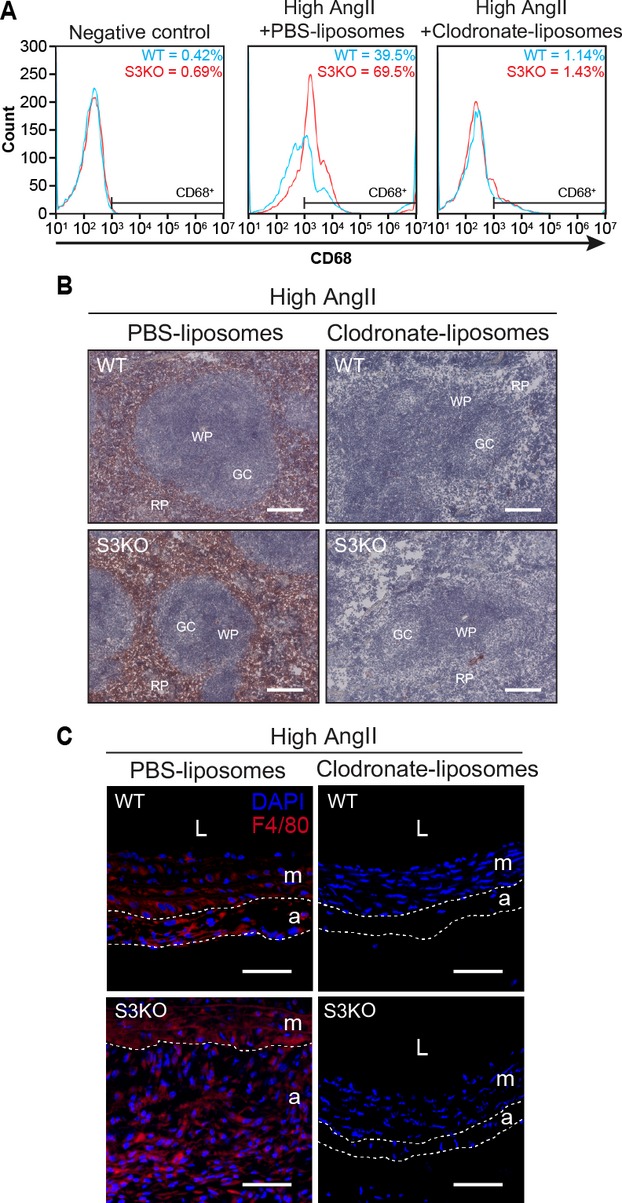
Macrophage‐depleting effect in mice after treatment with clodronate‐liposomes. A, Representative histograms generated from flow cytometry showing the percentage of circulating CD68^+^ monocytes in total white blood cells. B, Immunohistochemical analysis of F4/80^+^‐macrophages (brown‐staining cells) in the spleen biopsies obtained from high‐dose AngII‐infused WT and S3KO mice after PBS‐ or clodronate‐liposome treatment. Scale bars=50 μm. C, Immunofluorescence staining for mature macrophages (F4/80^+^ cells) in S3KO and WT aortas. Dotted lines demarcate adventitial layer from medial layer. Scale bars=50 μm. WT indicates wild type; S3KO, *SMAD3* knockout; PBS, phosphate‐buffered saline; AngII, angiotensin II; RP, red pulp; WP, white pulp; GC, germinal centers; L, lumen; m, tunica media; a, tunica adventitia.

**Figure 14.**
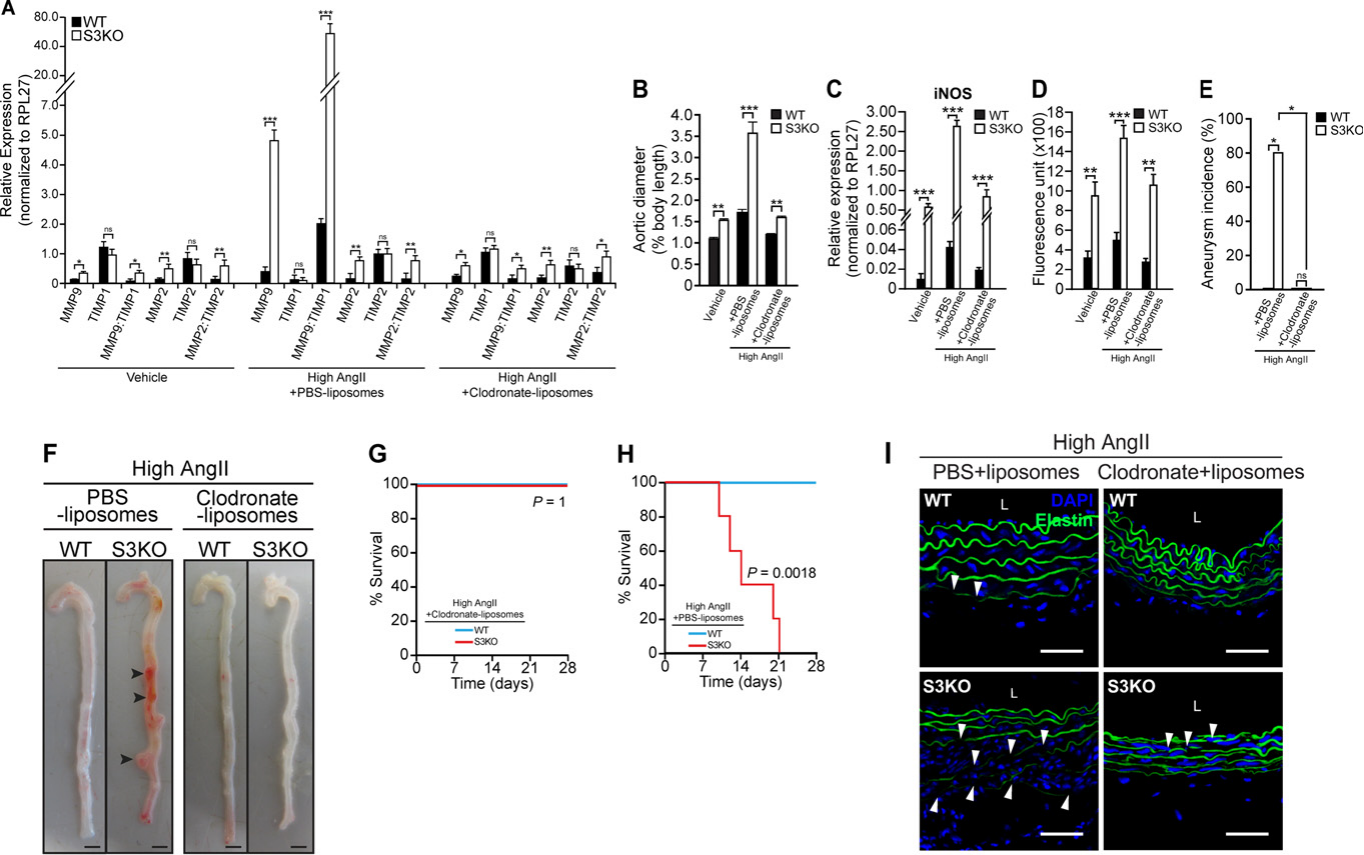
Clodronate‐liposomes abrogate aneurysm formation in AngII‐infused S3KO mice but fail to restore pristine state of elastin. A, Relative mRNA expression of MMP‐2, MMP‐9, and their respective inhibitors in vehicle‐ and AngII‐infused WT and S3KO aortas after PBS‐ or clodronate‐liposome treatment (n=3 per group; triplicate experiments were performed). Gene expression was normalized to RPL27. B, Changes in aortic diameter after PBS‐ or clodronate‐liposome treatment (n=5 per group). C, Relative mRNA expression of iNOS (n=3 per group; triplicate experiments were performed). D, Relative NO production presented in fluorescence units from S3KO and WT aortic sections (n=3 per group; triplicate measurements were performed). E, Aortic aneurysm incidence. F, Representative photographs showing the gross phenotype of high‐dose AngII‐infused WT and S3KO aortas after PBS‐ or clodronate‐liposome treatment. Arrowheads indicate aortic aneurysms/dissections. Scale bars=2 mm. G and H, Kaplan–Meier plot demonstrating the survival rate in high‐dose AngII‐infused S3KO mice after clodronate‐ (n=5 per group) (G) or PBS‐liposome (n=5 per group) (H) treatment. *P* values on the survival curves were generated using the log‐rank test after comparing with the respective WT counterparts. I, Immunofluorescence staining for elastin in the aorta. Arrowheads indicate elastic fiber fragmentations. Scale bars=50 μm. Values represent mean±SEM. *P<0.05, **P<0.01, ***P<0.001. AngII indicates angiotensin II; MMP, matrix metalloproteinase; WT, wild type; S3KO, *SMAD3* knockout; PBS, phosphate‐buffered saline; RPL27, ribosomal protein L27; iNOS, inducible nitric oxide synthase; TIMP, tissue inhibitor of metalloproteinases; L, lumen; ns, not significant.

To directly address the role of monocytes/macrophages in aneurysm development, we depleted circulating monocytes with clodronate‐containing liposomes in the high‐dose AngII‐infused mice (Figure 13A). Notably, clodronate‐liposomes eliminated almost all spleen and aortic F4/80^+^ macrophages (Figure 13B and 13C) while reducing aortic diameter, iNOS and MMP‐9 expressions, and NO production to levels comparable with the vehicle‐infused aortas (Figure 14A through 14D). Clodronate‐liposomes also completely abrogated aneurysm development and death from rupture in the high‐dose AngII‐infused S3KO mice. All S3KO mice (5 of 5) treated with PBS‐liposomes died from aortic rupture within 21 days of AngII infusion (Figure 14E through 14H). However, macrophage depletion failed to restore the elastic lamellae to their normal state, as the clodronate‐treated S3KO aortas still exhibited mild elastin fragmentation similar to that observed in vehicle‐infused aortas (Figure 14I, compare with Figure 6B). Together, these findings support the hypothesis that the macrophage‐mediated immune response is essential for the development of aneurysms, but VSMCs are responsible for the initial structural defects observed in S3KO mice.

### Aminoguanidine Treatment Completely Eliminates Aortic Aneurysm and Restores Elastin Content in AngII‐Infused S3KO Mice

**Figure 15.**
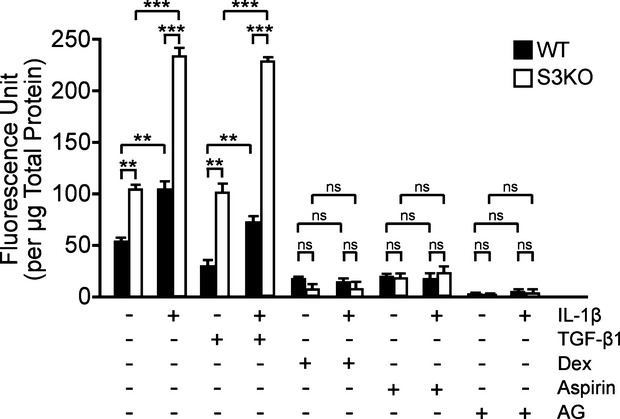
Effects of anti‐inflammatory drugs and iNOS‐selective inhibitor on iNOS‐derived NO production in *ex vivo* aortic segments. NO production in response to 10 ng/mL of TGF‐β1, 500 μmol/L of dexamethasone, 500 μmol/L of aspirin, or 500 μmol/L of aminoguanidine with or without the stimulation of 10 ng/mL IL‐1β in S3KO and WT endothelium‐denuded aortic segments. Values represent mean±SEM. **P<0.01, ***P<0.001. iNOS indicates inducible nitric oxide synthase; NO, nitric oxide; TGF‐β1, transforming growth factor‐β1; IL‐1β, interleukin‐1β; S3KO, *SMAD3* knockout; WT, wild type; Dex, dexamethasone; AG, aminoguanidine; ns, not significant.

To understand the role of inflammation in AngII‐induced aneurysm in S3KO mice, we first examined the *ex vivo* effects of TGF‐β1, steroidal and nonsteroidal anti‐inflammatory drugs (ie, dexamethasone and aspirin, respectively), and an iNOS‐selective inhibitor (ie, aminoguanidine) on iNOS‐mediated NO production in endothelium‐denuded aortic rings. In the untreated condition, S3KO aortas had significantly greater production of NO than did WT aortas; this increase was further exaggerated on IL‐1β stimulation (Figure 15). TGF‐β1 treatment reduced NO production in both unstimulated and IL‐1β‐stimulated WT aortas. This inhibitory effect was not observed in S3KO aortas with or without IL‐1β stimulation, indicating that SMAD3 is required for the inhibitory effects of TGF‐β1. Both dexamethasone and aspirin significantly reduced NO production in S3KO aortas to levels comparable to cognate WT aortas, suggesting that inflammation also affects iNOS‐mediated NO production. Treatment with aminoguanidine almost completely abolished NO production, reducing it to levels lower than those achieved with anti‐inflammatory treatment.

**Figure 16.**
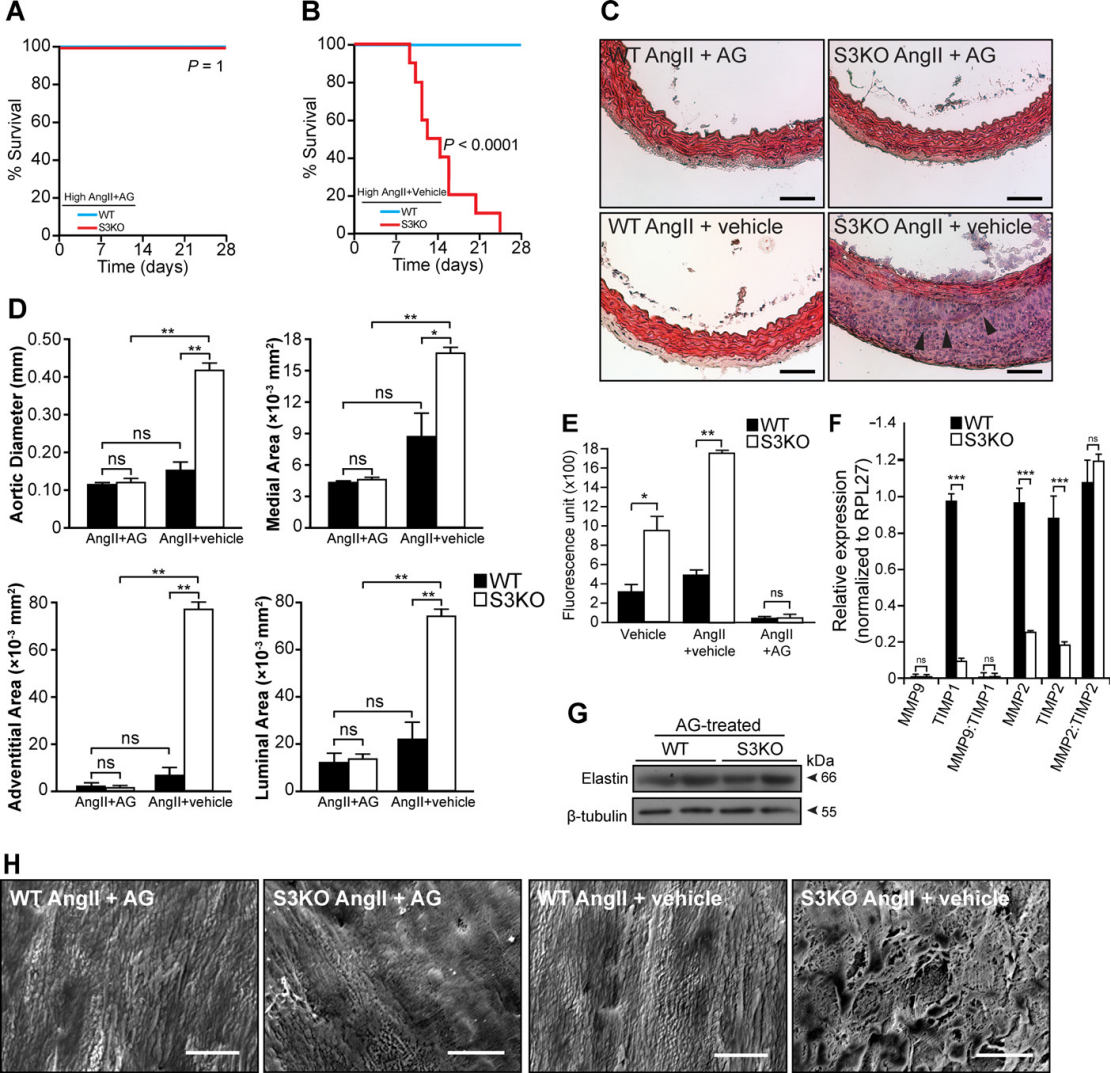
Aminoguanidine treatment abolishes aneurysm formation and restores elastin content in AngII‐infused S3KO mice. A and B, Kaplan–Meier plot demonstrating the survival rate in high‐dose AngII‐infused S3KO mice after aminoguanidine (n=10 per group) (A) or vehicle (n=10 per group) (B) treatment. *P* values on survival curves were generated using the log‐rank test after comparing S3KO mice with their respective controls. C and D, Hematoxylin and eosin staining (C) and morphometric analysis (D) of AngII‐infused aorta after aminoguanidine or vehicle treatment. Arrowheads indicate medial dissections. Scale bars=50 μm. E, Relative NO production from aortic sections presented in fluorescence units (n=3 per genotype; triplicate measurements were performed). F, Relative mRNA expression of MMP‐2, MMP‐9, and their respective inhibitors in AngII‐infused WT and S3KO aortas after aminoguanidine treatment (n=3 per genotype; triplicate experiments were performed). Gene expression was normalized to RPL27. G, Western blot analysis of aortic elastin content. Beta‐tubulin served as a loading control. H, Scanning electron microscopy of internal elastic lamella. Note that comparisons of histology and ultrastructure between S3KO and WT aortas were made on the basis of the same anatomical location. Scale bars=10 μm. Values represent mean±SEM. *P<0.05, **P<0.01, ***P<0.001. AngII indicates angiotensin II; S3KO, *SMAD3* knockout; NO, nitric oxide; WT, wild type; MMP, matrix metalloproteinase; RPL27; ribosomal protein L27; AG, aminoguanidine; TIMP, tissue inhibitor of metalloproteinases; ns, not significant.

Next, we administered aminoguanidine to the high‐dose AngII‐infused mice. In contrast to the vehicle‐treated AngII‐infused group, in which all S3KO mice (10 of 10) died within 24 days, all aminoguanidine‐treated S3KO and WT mice survived AngII infusion (Figure 16A and 16B). Moreover, aminoguanidine treatment completely abolished the occurrence of aortic aneurysms and dissections. Histologic analysis further confirmed that aminoguanidine treatment totally abrogated the medial dissections, aortic dilatation, medial and adventitial thickening, and luminal enlargement previously observed in the AngII‐infused S3KO aortas (Figure 16C and 16D). Immunofluorescence staining for NO also demonstrated a profound reduction of NO in the aminoguanidine‐treated groups compared with both vehicle‐ and AngII‐infused mice (Figure 16E). Furthermore, the MMP‐9 mRNA level and the MMP‐9:TIMP‐1 ratio were markedly downregulated in S3KO aortas after aminoguanidine treatment, and the significant differences between S3KO and WT mice disappeared (Figure 16F). Downregulation of MMP‐2 and its inhibitor TIMP‐2 was also observed in S3KO aortas. However, the overall MMP‐2:TIMP‐2 ratio remained unchanged in the 2 groups. Importantly, aminoguanidine treatment restored the elastin content and ultrastructural integrity of the internal elastin lamella in S3KO mice (Figure 16G and 16H). These observations confirm that aberrant iNOS‐mediated NO production is a driver, rather than a marker, of aortic aneurysm progression in S3KO mice.

## Discussion

Despite the identification of *SMAD3* mutations in human AOS,^4–5^ the molecular events leading to the pathogenesis of aortic aneurysms in these patients remain elusive. A well‐defined experimental model will be invaluable in advancing our knowledge of this multisystem connective tissue disorder. However, an animal model has not been identified. Furthermore, the etiopathogenesis of aortic aneurysms in these patients remains unknown. Here, we have demonstrated that AngII‐induced vascular inflammation, but not hypertension, triggers aortic aneurysm formation in S3KO mice. SMAD3 deficiency caused an intrinsic weakening of the aortic wall because of a deteriorated extracellular matrix involving the degradation of elastic lamella and collagen fibers. These primary defects alone were inadequate to cause aortic aneurysms in S3KO mice. In conjunction with AngII‐induced vascular inflammation mediated by the enhanced transmural infiltration of macrophages, these abnormalities predisposed S3KO mice to the development of highly penetrant and severe aortic aneurysms, recapitulating the pathologic phenotypes of human AOS.^4–5^ We further revealed that SMAD3 deficiency caused enhanced iNOS‐mediated NO production to predominantly activate elastolytic and collagenolytic MMP‐9 in the VSMCs, which in turn contributed to the initial structural defects observed in S3KO aortas prior to AngII treatment. AngII‐induced vascular macrophage recruitment further exacerbated the pathological effects of the iNOS‐NO‐MMP‐9 cascade to promote aortic aneurysm formation and progression in S3KO mice. Macrophage depletion in AngII‐infused S3KO mice only partially reduced the iNOS‐mediated NO production and MMP‐9 expression, bringing them close to levels seen in the untreated mice. Although this strategy prevented aneurysm formation, mild elastin fragmentation still persisted in S3KO aortas. In contrast, treatment with an iNOS inhibitor completely abolished the iNOS‐NO‐MMP‐9 cascade in these mice and restored the integrity of the elastic lamella. Altogether, this work provides a mechanistic insight into the etiopathogenesis of aortic aneurysms in human AOS and underscores a crucial role for macrophage‐mediated immune response in disease progression.

Apart from its vasopressor effect, AngII has been known to trigger vascular inflammation via the activation of NF‐κB and to enhance the transcription of proinflammatory cytokines (eg, MCP‐1).^37^ We showed that hypertension serves as a risk factor for aortic rupture, whereas vascular inflammation alone could trigger aneurysm formation in S3KO mice. Consistent with these findings, enhanced phosphorylation of NF‐κB and expression of MCP‐1 were detected in AngII‐infused S3KO mice. The increased transmural recruitment of macrophages further exacerbates the iNOS‐NO‐MMP‐9 cascade to accelerate matrix degradation. In addition, SMAD3 has been shown to directly regulate TGF‐β1‐mediated inhibition of MCP‐1 and vascular inflammation,^36^ congruent with the findings that TGF‐β activity protects against inflammatory aortic aneurysm progression and complications in AngII‐induced mice.^23^ Indeed, mice lacking MCP‐1 exhibit a reduction in cerebral aneurysm formation and macrophage accumulation accompanied by decreased expression of iNOS, MMP‐2, and MMP‐9.^30^ We also showed for the first time that LPS‐induced vascular inflammation plays a causative role in aneurysm initiation, despite that LPS has previously been detected in aneurysmal arterial walls and has been demonstrated to induce aortic iNOS expression.^38–39^ Although anti‐inflammatory drugs may represent an attractive candidate for the treatment of aortic aneurysms in S3KO mice, our *ex vivo* data highlighted that iNOS inhibition has a more effective and direct impact on the production of iNOS‐derived NO, parallel with the finding that macrophage depletion failed to prevent elastin fragmentation.

NO produced by iNOS plays a pivotal role in the pathogenesis of inflammation and aneurysm.^40–41^ Increased iNOS expression has been implicated in rodent and human aortic aneurysms.^28–29^ Inhibition of iNOS has yielded positive outcomes in limiting aneurysm expansion in an elastase‐induced aneurysm model and in the prevention of cerebral aneurysm formation in a hypertensive rat model.^25,40–41^ In agreement with these findings, we revealed that iNOS‐derived NO has a detrimental effect on aortic structural integrity in S3KO mice. Although activated macrophages are most frequently responsible for iNOS expression, VSMCs have also been shown to produce this enzyme after stimulation with proinflammatory cytokines,^42^ which is consistent with our observation that both NO and iNOS staining were localized in the medial layer of untreated S3KO aortas, suggesting that VSMCs were initially involved in iNOS‐mediated NO production. Interestingly, NO‐mediated monocyte chemotaxis has also been reported,^43^ suggesting that enhanced iNOS‐derived NO production in VSMCs may represent an early event preceding the influx of inflammatory macrophages into the aortic wall. Given that the induction of iNOS has been shown to increase the production and activation of MMP‐9 in atheromas in *APOE*‐knockout mice,^34^ we observed drastic elevation in the expression and activation of MMP‐9 along with enhanced iNOS‐derived NO production in S3KO aortas. Indeed, NO‐regulated MMP‐9 secretion from murine macrophages can occur via several mechanisms, as previously described.^44^ Data from animal studies have shown that MMP‐2 and MMP‐9, which are predominantly secreted by macrophages,^45^ work in concert to produce aortic aneurysms.^46^ In addition, the targeted gene disruption of MMP‐9 suppresses the development of aortic aneurysms, thereby providing direct evidence that MMP‐9 plays an essential role in nonatherosclerotic aortic aneurysm formation.^47^ Increased serum levels of MMP‐9 have also been implicated in the pathogenesis of coronary artery aneurysms in Kawasaki disease, a multisystemic vasculitis mostly found in children.^48^ Generally, aortic dilatation requires elastolysis, whereas vessel wall rupture depends on collagenolysis.^49^ Interestingly, MMP‐9 possesses both elastolytic and collagenolytic properties,^46,50^ confirming its roles in aneurysm formation and rupture. Moreover, MMP‐2 has demonstrated activity against intact collagen fibrils and soluble native type I collagen fibrils,^51^ whereas MMP‐9 has activity against soluble, monomeric forms of native collagen types I and III.^50^

The vascular inflammatory response involves complex interactions among the recruited inflammatory cells (eg, lymphocytes, macrophages, and neutrophils), vascular resident cells (eg, endothelial cells, VSMCs, and adventitial fibroblasts), and the extracellular matrix.^52^ MCP‐1 secreted by VSMCs and adventitial fibroblasts is believed to be an important mediator in the early pathogenesis of aortic aneurysms.^52^ MCP‐1 induces monocyte chemotaxis by binding to CC‐chemokine receptor 2 (CCR2), whereas CCR2 deficiency prevents progressive aortic expansion in various mouse models.^53–54^ In addition, macrophages express 5‐lipooxygenase, which produces macrophage inflammatory protein‐1α to recruit T cells in a paracrine fashion.^55^ Infiltrated T cells further contribute to the proinflammatory cytokine storm at the aneurysmal aortic wall. Interestingly, many proinflammatory cytokines, such as IL‐1β, granulocyte‐macrophage colony‐stimulating factor, tumor necrosis factor‐α, and interferon‐γ, have been reported in human abdominal aortic aneurysms and can induce the expression of iNOS via NF‐κB and C/EBP.^33,56–63^ We also recognized that the antianeurysm effects of aminoguanidine may not be entirely attributed to iNOS inhibition, as it has also been shown to act as a scavenger for advanced glycation end products and reactive oxygen species, which are also known to promote the development of aortic aneurysms.^64–66^ Thus, we could not eliminate the possibility that other cellular and molecular components may also contribute to the development of aortic aneurysms in our system. Further studies using SMAD3 and iNOS double‐knockout mice and tissue‐specific knockout of iNOS will unequivocally validate the therapeutic effects of iNOS deficiency in our model.

To date, surgery is still the definitive treatment for aneurysms, whereas therapy involving strict blood pressure control is typically supportive. Based on our in vivo data, iNOS antagonism represents a promising approach to tackling aortic aneurysms related to *SMAD3* mutations, and it merits further investigation as an adjunctive strategy for AOS patients to prevent major life‐threatening manifestations of this disorder. In light of the pathologic phenotypes (eg, aortic aneurysms and early‐onset osteoarthritis) common between S3KO mice and human AOS, we posit that these mice represent a legitimate animal model for the elucidation of the molecular mechanisms leading to other pathologic manifestations of AOS.

## Author Contributions

Dr Chek Tan carried out the experiments, analyzed and prepared data for publication, and wrote the manuscript. Drs Eddie Tan, Luo, Huang, Loo, and Choong conducted parts of the experiments. Dr Nguan Tan revised the manuscript and supervised the project.
